# *Eupatorium lindleyanum* DC Ameliorates Carbon Tetrachloride-Induced Hepatic Inflammation and Fibrotic Response in Mice

**DOI:** 10.3390/ph18081228

**Published:** 2025-08-20

**Authors:** Jinbao Yang, Yufei Wang, Lijuan Zhuo, Guijun Lu, Meiting Zhang, Jiabin Huang, Yehaomin Li, Wenwen Liu, Jing Qi, An Zhu, Zixiong Zhou

**Affiliations:** 1 Department of Pathology & Institute of Oncology, School of Basic Medical Sciences, Fujian Medical University, Fuzhou 350122, China; yangjinbao@stu.fjmu.edu.cn (J.Y.); zhuolijuan@fjmu.edu.cn (L.Z.); luguijun@stu.fjmu.edu.cn (G.L.); zhangmeiting@stu.fjmu.edu.cn (M.Z.); a15079433673@fjmu.edu.cn (J.H.); liyehaomin@stu.fjmu.edu.cn (Y.L.); wenzi0538@fjmu.edu.cn (W.L.); 2 Department of Biochemistry and Molecular Biology, School of Basic Medical Sciences, Fujian Medical University, Fuzhou 350122, China; 2230130025@fjmu.edu.cn (Y.W.); qijing2020@fjmu.edu.cn (J.Q.); 3 Key Laboratory of Gastrointestinal Cancer, Fujian Medical University, Ministry of Education, Fuzhou 350108, China

**Keywords:** liver fibrosis, *Eupatorium lindleyanum* DC, inflammation, PDGF/PDGFR-β signaling, hepatic stellate cell

## Abstract

**Background/Objectives:** *Eupatorium lindleyanum* DC (Eup), a traditional Chinese medicinal herb, is widely used for treating inflammation-mediated diseases, including pneumonia. However, its potential therapeutic effects on inflammation-driven liver fibrosis remain to be elucidated. This study aimed to investigate the effects of Eup on carbon tetrachloride (CCl_4_)-induced liver fibrosis and elucidate its underlying mechanisms. **Methods:** The chemical constituents of Eup were analyzed using ultra-performance liquid chromatography coupled with quadrupole time-of-flight mass spectrometry (UPLC-Q/TOF-LC/MS). A CCl_4_-induced liver fibrosis murine model and LX-2 cells were used in study. Serum biochemical assays, histological analysis, qRT-PCR, ELISA, and Western blot were used to assess Eup’s anti-inflammatory and anti-fibrotic properties. RNA sequencing (RNA-seq) was employed to identify potential mechanisms, with validation by Western blot. **Results:** 89 and 49 compounds were identified in Eup under positive and negative ion modes, respectively. *In vivo*, Eup treatment decreased collagen deposition and expression levels of fibrosis-related genes, including collagen I and α-smooth muscle actin. Additionally, Eup alleviated hepatic inflammation. *In vitro*, Eup inhibited FBS-induced hepatic stellate cell (HSCs) activation. Gene set enrichment analysis (GSEA) indicated that Eup significantly downregulated the platelet-derived growth factor (PDGF)/platelet-derived growth factor receptor-beta (PDGFR-β) signaling pathway, which was further validated in both CCl_4_-induced fibrotic livers and PDGF-BB-activated HSCs using western blot. **Conclusions:** Eup attenuated liver fibrosis by inhibiting inflammation and suppressing HSCs activation via downregulating PDGF/PDGFR-β signaling pathway.

## 1. Introduction

Hepatic fibrosis, a common pathological outcome of chronic liver disease (CLD), is marked by excessive extracellular matrix (ECM) deposition and abnormal scar tissue formation [1]. It is primarily triggered by persistent liver injury caused by factors such as chronic hepatitis B or C infection, alcohol abuse, metabolic dysfunction-associated steatotic liver disease (MASLD), and cholestasis [2,3]. Without effective intervention, liver fibrosis can progress to irreversible cirrhosis and hepatocellular carcinoma (HCC), significantly impairing patient prognosis and quality of life. Globally, liver-related diseases account for approximately 2 million deaths annually, representing 4% of total mortality, with cirrhosis and HCC being the leading causes [4]. Despite its significant health burden, the molecular mechanisms underlying liver fibrosis remain incompletely understood, and there is a pressing need for safe and effective therapeutic agents to halt or reverse its progression.

Liver fibrosis, resulting from CLD, represents a reparative response characterized by the excessive and disorganized accumulation of collagen produced by activated HSCs and their myofibroblast derivatives [5]. In the context of repeated injury, damaged hepatocytes, infiltrating immune cells, resident macrophages, and HSCs secrete various paracrine and autocrine growth factors and inflammatory chemokines, perpetuating a cycle of tissue damage and remodeling that results in the formation of a fibrotic matrix. The injury to hepatocytes initiates an inflammatory reaction, activating macrophages, releasing reactive oxygen species (ROS), transforming growth factor-beta 1 (TGFβ1) [6], and converting dormant HSCs into myofibroblast-like cells [7]. Once activated, HSCs/myofibroblasts proliferate in response to cytokines such as PDGF, express fibrogenic markers like α-SMA, secrete type I collagen, and contribute to liver fibrosis [8]. When the underlying cause of injury is resolved, myofibroblasts either undergo apoptosis or revert to an inactive state. Therefore, inhibiting the activation of HSCs or eliminating fibrogenic myofibroblasts are key therapeutic approaches to prevent the progression of fibrosis [9]. Targeting liver lipid metabolism, oxidative stress, inflammation, and cell death may represent an alternative therapeutic approach for hepatic fibrosis [10].

Several key pathways contribute to HSC activation, including TGF-β signaling pathway, PDGF signaling pathway, Hippo signaling pathway, and ROS [11]. Among these, the PDGF signaling pathway exhibits specificity for HSCs. The PDGF family comprises four ligands (PDGF A–D) that signal through dimeric transmembrane receptors, PDGFR-α, and PDGFR-β [12]. While PDGFR-α expression remains stable in both quiescent and activated HSCs, PDGFR-β expression correlates with the severity of liver fibrosis [13,14]. Ligand binding induces receptor dimerization and tyrosine phosphorylation, activating downstream pathways such as p38 MAPK, PI3K/AKT, and Ras/tyrosine kinase, which promote HSCs’ proliferation and survival [15,16,17]. Notably, genetic depletion of PDGFR-β in HSCs has been shown to attenuate liver injury and fibrosis *in vivo* [18]. Enhanced PDGF-BB secretion by L02 cells has been shown to promote LX-2 cell activation in co-culture systems [19]. Furthermore, compounds such as Salvianolic acid B [20], Roseotoxin B [21], and Gomisin D [22] have demonstrated anti-fibrotic effects by targeting the PDGF/PDGFR-β pathway, underscoring its therapeutic potential.

*Eupatorium lindleyanum* DC (Eup) (plant name was checked at http://www.theplantlist.org), commonly known as “Yemazhui”, is a traditional Chinese medicine herb classified under the genus Eupatorium in the Compositae family, widely distributed in China, with Jiangsu Province being its authentic producing area [23,24]. Historically, Eup has been used by local communities to treat respiratory conditions such as cough and tracheitis [25]. More than 100 bioactive ingredients have been identified in Eup, including triterpenoids, sesquiterpenes, diterpenoids, organic acids, flavonoids, volatile oils, and amino acids [26]. Substantial differences in chemical composition were observed across distinct medicinal parts [27]. Eup is traditionally recognized for its properties of relieving cough, dispelling phlegm, clearing heat, and detoxifying [28]. It has garnered attention for its anti-inflammatory [25,29], anti-tumor [30,31], and antioxidant effects [29,32]. However, its potential role in hepatic fibrosis remains unexplored.

In this study, we aimed to investigate the anti-fibrotic efforts of Eup and elucidate its underlying mechanism. To investigate this, we utilized a CCl_4_-induced mouse model of liver fibrosis and the LX-2 human hepatic stellate cell line to assess the pharmacological impact of Eup. RNA sequencing was employed to identify the pathways modulated by Eup. Our findings aim to offer a scientific foundation for advancing Eup as a novel therapeutic agent for liver fibrosis, bridging traditional medicinal use with modern pharmacological evidence.

## 2. Results

### 2.1. Chemical Profiling of Eup Extract

**Figure 1:**
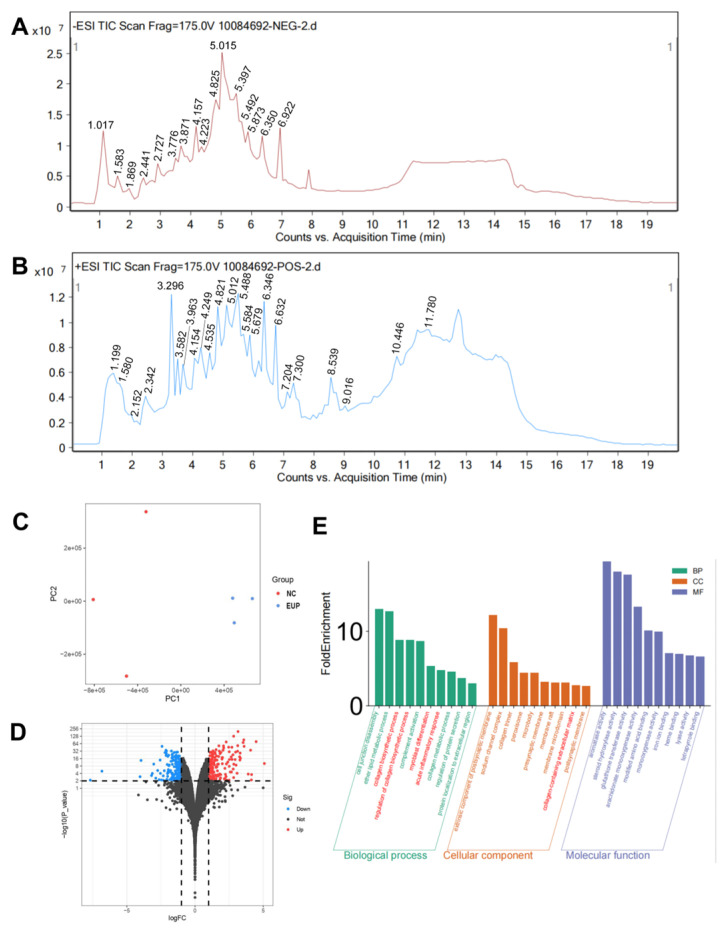
Chemical profiling of Eup extract and transcriptomic analysis of RNA sequencing data. (**A**) Representative UPLC-Q/TOF-LC/MS total ion chromatogram of Eup obtained in negative ionization mode. (**B**) Corresponding UPLC-Q/TOF-LC/MS total ion chromatogram of Eup acquired in positive ionization mode. (**C**) PCA of RNA-seq data from the NC group and the 40 g/kg Eup group (n = 3 per group). (**D**) Volcano plot visualization of DEGs comparing the NC group and the 40 g/kg Eup-treated group. Significantly upregulated genes are denoted by red data points, while downregulated genes are indicated by blue data points. (n = 3 per group; DEGs with adjusted *p* < 0.05). (**E**) Heatmap and hierarchical clustering showing the separation of the NC group and the 40 g/kg Eup group, n = 3 per group.

The chemical constituents of the Eup extract were analyzed using UPLC-Q/TOF-LC/MS. In total, 89 distinct compounds were characterized in positive ion mode, while 49 compounds were identified under negative ion detection conditions (Figure 1A,B). The detailed components were listed in Table 1 and Table 2. Among these components, Citric acid can improve LPS-induced liver injury [33], and Embelin demonstrated protective effects against thioacetamide-induced acute hepatic injury in murine models [34]. Moreover, a recent study has shown that oxalic acid can protect cells from oxidative stress [35]. Based on the presence of these hepatoprotective components, Eup may have an ameliorative effect on liver fibrosis.

### 2.2. Bioinformatics Analysis of mRNA-Seq Gene

RNA sequencing analysis was performed on liver tissues from the normal control group (n = 3) and the 40 g/kg Eup group (n = 3) using the Illumina NovaSeq 6000 platform (Illumina, San Diego, CA, USA). PCA of gene expression profiles revealed distinct clustering patterns between the control group and the 40 g/kg Eup group (Figure 1C). Similarly, samples from the control group and the 40 g/kg Eup group were separately clustered in a Clustered Heatmap (Figure S3). Differential expression analysis identified 1294 significantly regulated genes (|log2FC| > 1, FDR < 0.05), comprising 704 upregulated and 590 downregulated genes in the 40 g/kg Eup group compared to controls (Figure 1D). Gene Ontology (GO) enrichment analysis of these differentially expressed genes demonstrated significant enrichment in biological processes, including collagen biosynthetic process, regulation of collagen biosynthetic process, myoblast differentiation, and acute inflammatory response (Figure 1E). These enrichment terms are highly associated with fibrogenesis, suggesting Eup may possess potential therapeutic efficacy in ameliorating hepatic fibrosis.

### 2.3. Eup Attenuates CCl_4_-Induced Liver Fibrosis in Mice

**Figure 2:**
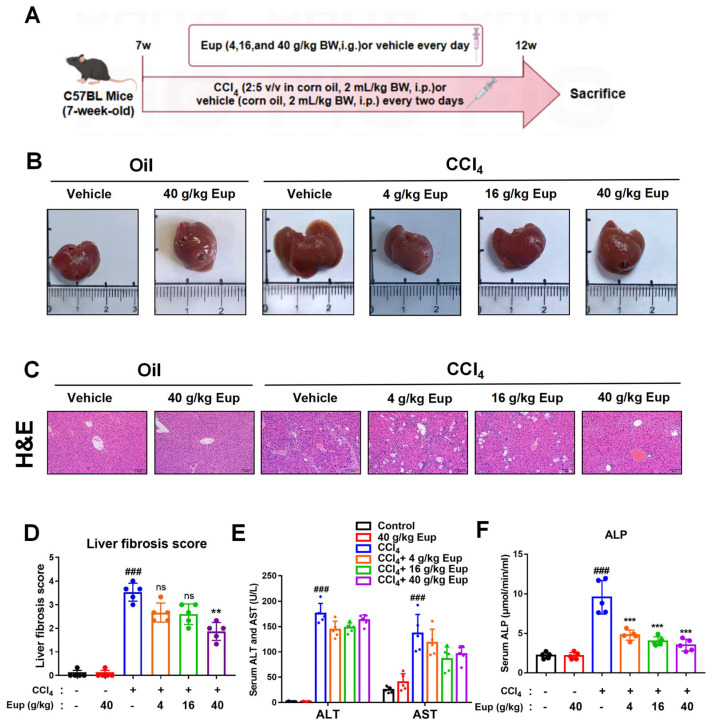
The potential of Eup to improve the murine liver fibrosis induced by CCl_4_. (**A**) Flow chart of CCl_4_-treated mice treated with gavage Eup or drinking water. Seven-week-old mice were intraperitoneally injected with CCl_4_ (2:5 *v*/*v* in corn oil) or the same volume of corn oil every two days. Each group was gavaged with Eup (4, 16, and 40 g/kg) or vehicle daily. After 5 weeks of treatment, the livers were collected from these mice. (**B**) Morphological observation of mouse livers. (**C**,**D**) H&E staining was performed to assess histological alterations in liver tissue sections across experimental groups. Representative views and the quantification results are presented; scale bar, 50 μm. (**E**,**F**) Serum concentrations of ALT, AST, and ALP were quantified. The statistical significances of different grades were defined as ^###^ *p* < 0.001 versus control group; ** *p* < 0.01 and *** *p* < 0.001 versus CCl_4_ group. The “ns” indicates non-significant (*p* ≥ 0.05).

To assess the anti-fibrotic effects of Eup, seven-week-old mice were intraperitoneally injected with CCl_4_ (2:5 *v*/*v* in corn oil) or the same volume of corn oil every two days. Meanwhile, each group was administered Eup (4, 16, and 40 g/kg) or vehicle daily (Figure 2A). Body weight measurements (initial and final), food consumption data, and hepatic weight parameters are presented in Supplementary Figure S1A–E. The liver index in the model group was significantly higher compared to the control group. Macroscopic examination revealed distinct morphological differences between groups (Figure 2B): control livers exhibited normal reddish coloration and smooth surfaces, whereas CCl_4_-treated livers displayed dark discoloration and coarse granular surfaces. Notably, 16 g/kg and 40 g/kg Eup treatment groups showed marked improvement in liver color and surface texture. Histopathological analysis by H&E staining demonstrated characteristic features of liver fibrosis in the CCl_4_ model group, including extensive hepatocyte necrosis, inflammatory cell infiltration, disruption of hepatic cord architecture, and portal/perisinusoidal fibrosis (Figure 2C). Quantitative fibrotic scoring revealed significant attenuation of these pathological changes in Eup-treated groups (Figure 2D). Consistent with histological findings, the level of serum ALT and AST in Eup treatment groups exhibited a decreased trend (Figure 2E), while the level of serum ALP was significantly decreased after Eup treatment (Figure 2F).

**Figure 3:**
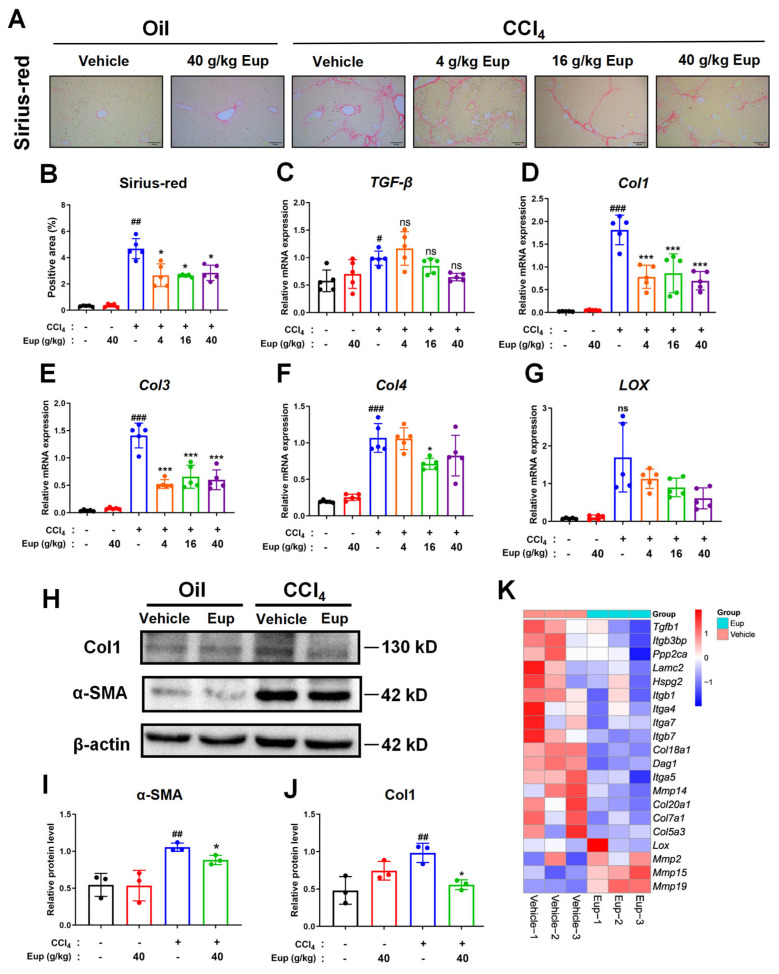
Eup attenuates CCl_4_-induced liver fibrosis in mice. (**A**,**B**) Sirius Red staining of liver tissues in different groups and the quantification results. Original magnification: × 200. (**C**–**G**) Relative mRNA expression levels of fibrosis-associated markers (*Col1*, *Col3*, *Col4*, *LOX*, and *TGF-β*) in liver tissues, normalized to *GAPDH* and analyzed by qRT-PCR (n = 5 per group). (**H**–**J**) Protein expression levels of α-SMA and Col1 in liver cells were analyzed using Western blot. (**K**) Heatmap of fibrotic gene expression in the CCl_4_-induced mice liver treated with Eup or vehicle. Data are expressed in the format of mean ± SD. The statistical significances of different grades were defined as ^#^ *p* < 0.05, ^##^ *p* < 0.01 and ^###^ *p* <0.001 versus control group; * *p* < 0.05 and *** *p* < 0.001 versus CCl_4_ group. The “ns” indicates non-significant (*p* ≥ 0.05).

Consequently, we aim to conduct further investigations into the therapeutic potential of Eup in treating CCl_4_-induced hepatic fibrosis. Sirius-red staining indicated a notable reduction of collagen deposition, with the improvement of the formation of pseudolobules after the administration of Eup. (Figure 3A,B). The qRT-PCR analysis revealed significantly elevated mRNA expression levels of *Col1*, *Col3*, *Col4*, *LOX*, and *TGF-β* in the model group. Notably, administration of medium and high doses of the treatment resulted in marked downregulation of these fibrotic markers, with *Col1*, *Col3*, and *Col4* demonstrating particularly significant reductions (Figure 3C–G). Protein expression analysis through Western blot corroborated these observations at the translational level, showing that Eup (40 g/kg) treatment significantly suppressed CCl_4_-induced upregulation of α-SMA and collagen I (Figure 3H–J). These proteins are well-established markers of HSC activation and fibrogenesis [36]. The transcriptomic analysis further supported these observations, with hierarchical clustering revealing the downregulation of fibrogenic genes (*Col1a1*, *Timp1*, *Tgfbr1*) and upregulation of matrix degradation genes (*Mmp2*, *Mmp9*) in the 40 g/kg Eup group compared to the control group (Figure 3K). These findings indicate that Eup has anti-fibrotic effects in the murine model.

### 2.4. Eup Treatment Therapeutically Mitigates the Inflammation Progression of CCl_4_-Evoked Hepatic Fibrosis

**Figure 4:**
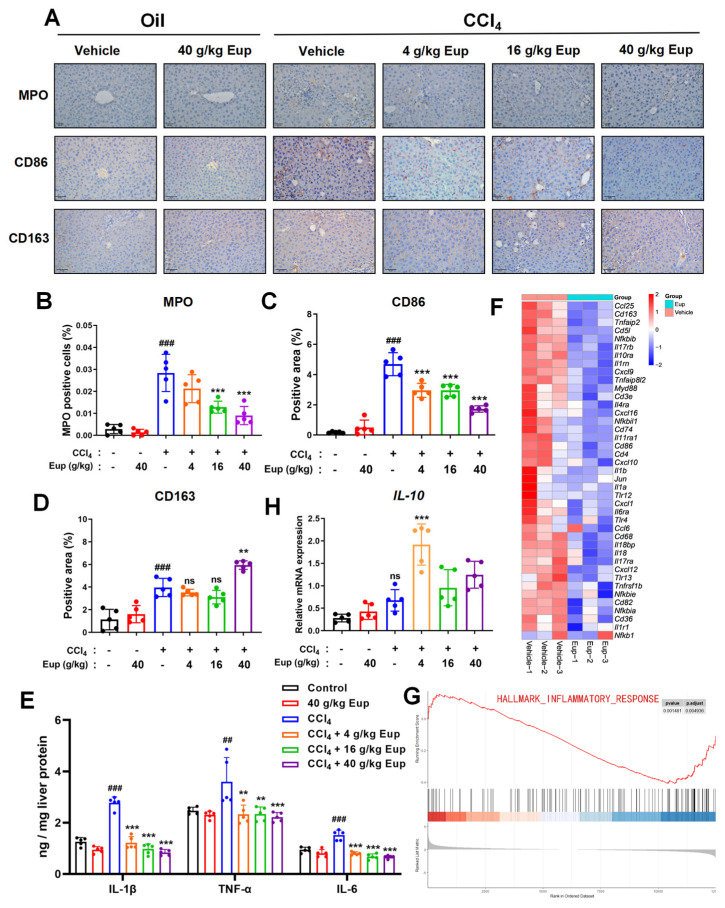
Eup treatment therapeutically mitigates the inflammation progression in liver tissues. (**A**) Immunohistochemistry of MPO, CD163, and CD86 in liver tissues from different groups, with each group administered different concentrations of Eup. Original magnification: ×200. (**B**–**D**) Quantitative analysis of immunohistochemically stained areas was performed using ImageJ software, with representative microscopic fields presented (scale bars: 200 μm for MPO, 50 μm for CD163 and CD86 immunohistochemistry). (**E**) Hepatic concentrations of pro-inflammatory cytokines, including IL-1β, TNF-α, and IL-6, were determined by ELISA. (**F**) Heatmap analysis showing the different levels of inflammatory factors between the vehicle group and the 40 g/kg Eup group was done (n = 3 per group). (**G**) GSEA analyses of the inflammatory response between the control group and the 40 g/kg Eup group. (**H**) Relative mRNA expression levels of *IL-10* in liver tissues, normalized to *GAPDH* and analyzed by qRT-PCR (n = 5 per group). Data are presented as means ± SD, n = 5 per group. Experimental groups marked by different signs represent significant differences between groups at *p* < 0.05. And the statistical significances of different grades were defined as ^##^ *p* < 0.01 and ^###^ *p* < 0.001 versus control group; ** *p* < 0.01 and *** *p* < 0.001 versus CCl_4_ group. The “ns” indicates non-significant (*p* ≥ 0.05).

To figure out the function of Eup in hepatic inflammation, the immunohistochemical staining method was applied. There was an increase in the number of CD86 + macrophages or MPO + neutrophils in CCl_4_-induced fibrotic livers in comparison with the untreated group, which was remarkably converted by Eup administration in a dose-dependent manner (Figure 4A–C). Consistent patterns were detected from pro-inflammatory mediators, specifically TNF-α, IL-1β, and IL-6, through ELISA quantification (Figure 4E). Moreover, the number of CD163 + macrophages was increased in the fibrotic liver after Eup treatment (Figure 4A,D). The mRNA expression levels of *IL-10*, an anti-inflammatory factor, were significantly upregulated after Eup treatment (4 g/kg) (Figure 4H). Additionally, Transcriptomic heatmap analysis confirmed systemic downregulation of inflammatory mediators in the Eup group (Figure 4F). Consistently, GSEA further revealed significant inhibition of key inflammatory pathways. Similarly, the regulation of lymphocyte activation and positive regulation of cytokine production were downgraded as well after administering Eup (Figure 4G and Figure S1G–I). All of these experimental data imply that Eup can significantly attenuate the hepatic inflammatory response in CCl_4_-induced fibrotic livers in mice.

### 2.5. Eup Treatment Inhibits HSC Activation In Vitro

**Figure 5:**
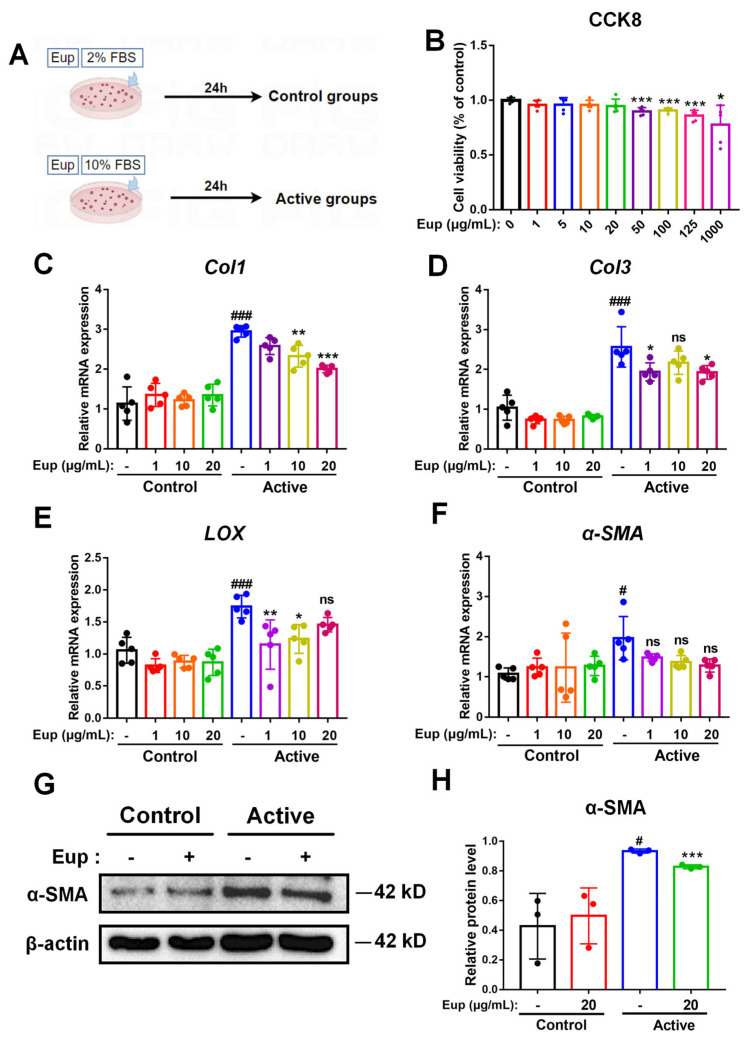
Eup treatment suppresses HSC activation *in vitro*. (**A**) Overview of the *in vitro* experiment. (**B**) The impact of Eup on cellular viability in quiescent LX-2 cells was evaluated using the CCK-8 assay (n = 5 per group). Data are normalized to untreated controls (0 μg/mL Eup). (**C–F**) The mRNA expression profiles of hepatic fibrotic markers, including *Col1*, *Col3*, *LOX*, and *α-SMA*, were analyzed using qRT-PCR. (**G**) Representative western blot bands of α-SMA and β-actin. (**H**) Protein expression of α-SMA was evaluated through Western blot analysis, with β-actin serving as the internal loading control. Quantitative densitometric analysis was performed using ImageJ software. Data are presented as means ± SD per group. The statistical significances of different grades were defined as ^#^ *p* < 0.05 and ^###^ *p* < 0.001 versus the control group; * *p* < 0.05, ** *p* < 0.01, and *** *p* < 0.001 versus the active group. The “ns” indicates non-significant (*p* ≥ 0.05).

To evaluate the impact of Eup on HSC activation, an *in vitro* experimental model was established through 24-h incubation of the LX-2 cells with 10% FBS (Figure 5A). CCK-8 assay revealed that the maximum safe concentration of Eup was 20 μg/mL in LX-2 cells (Figure 5B). To assess the therapeutic effect of Eup at different concentrations on HSCs activation, intracellular mRNA expression was quantitatively examined by qRT-PCR analysis. As shown in Figure 5C–F, the mRNA levels of fibrosis-related indicators, including *α-SMA*, *Col1*, *Col3*, and *LOX*, in LX-2 cells were significantly increased after incubating with 10% FBS, indicating that 10% FBS can significantly induce HSC activation. Nevertheless, treating with Eup decreased the mRNA levels of Col1, Col3, and LOX significantly. Thereinto, the mRNA expression of Col1 declined in a dose-dependent manner. While qRT-PCR analysis showed a modest decrease in α-SMA mRNA expression, Western blot quantification demonstrated significant downregulation of α-SMA protein expression with Eup administration (Figure 5G,H). Taken together, these results provide compelling evidence that Eup exerts potent inhibitory effects on HSC activation under *in vitro* conditions.

### 2.6. Eup Improves CCl_4_-Mediated Murine Liver Fibrosis by Modulating the PDGF/PDGFR-β Signaling Pathway

**Figure 6:**
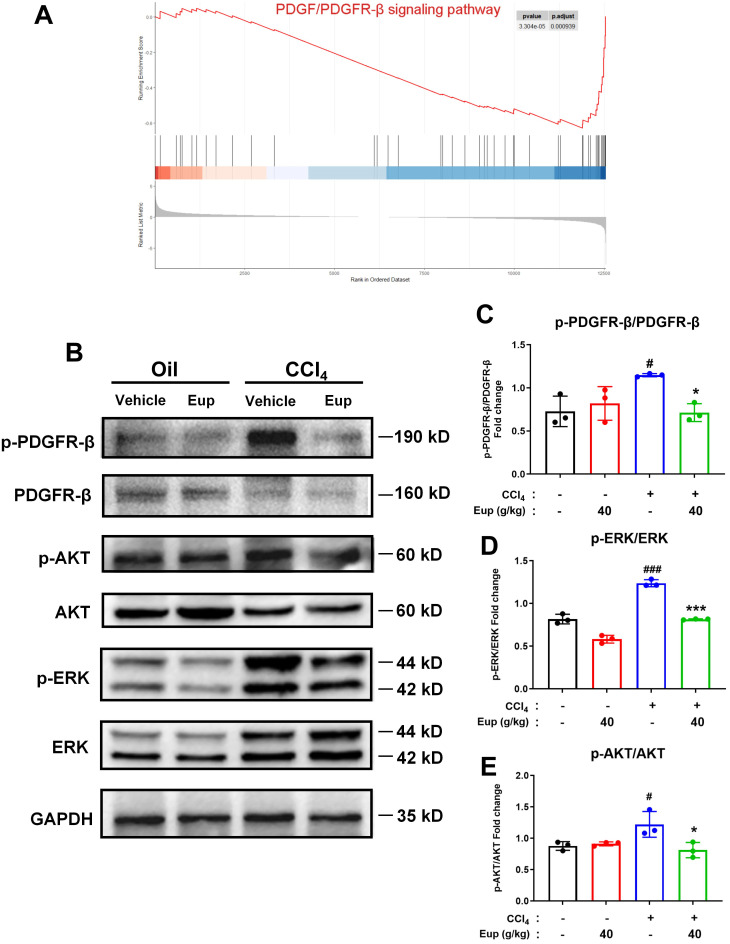
Eup exerts anti-fibrotic effects by attenuating CCl_4_-induced liver fibrosis through modulation of the PDGF/PDGFR-β signaling pathway. (**A**) GSEA analyses of PDGF/PDGFR-β signaling pathway between the NC group and the 40 g/kg Eup group (n = 3 per group). (**B**) Representative immunoblot images demonstrating protein expression of GAPDH and PDGF-BB/PDGFR-β signaling pathway-related proteins. (**C**–**E**) The activation status of the PDGF-BB/PDGFR-β signaling pathway-related proteins was quantified through densitometric analysis using Image J (p-PDGFR-β/PDGFR-β, p-AKT/AKT, and p-ERK/ERK). GAPDH was the loading control. Data are presented as mean ± SD, n = 3. ^#^ *p* < 0.05 and ^###^ *p* < 0.001 versus control group; * *p* < 0.05 and *** *p* < 0.001 versus CCl_4_ group.

RNA-sequencing analysis revealed that treatment with Eup (40 g/kg) significantly downregulated the PDGF/PDGFR-β signaling pathway in hepatic tissues (Figure 6A). To further validate these findings, we assessed the phosphorylation levels of PDGFR-β in liver tissues via Western blotting. Our findings revealed that the expressions of p-PDGFR-β/PDGFR-β proteins were significantly higher than those in the normal control group after CCl_4_ treatment, whereas administration of 40 g/kg Eup significantly downregulated the ratio of p-PDGFR-β/PDGFR-β proteins in the liver fibrosis model (Figure 6B,C). These results preliminarily indicate that Eup may ameliorate CCl_4_-evoked liver fibrosis by modulating the PDGF/PDGFR-β signaling pathway. To further elucidate the downstream mechanisms, we evaluated the PI3K/AKT and MAPK pathways (ERK). CCl_4_ exposure significantly increased the p-AKT/AKT and p-ERK/ERK protein ratios compared to the control group. However, Eup administration (40 g/kg) reversed these effects, normalizing both AKT and ERK phosphorylation levels (Figure 6B,D,E). These results suggested that Eup suppressed the downstream signaling pathways of PDGFR-β. Therefore, Eup can ameliorate murine liver fibrosis triggered by CCl_4_ through modulating the PDGF/PDGFR-β signaling pathway.

### 2.7. Eup Suppresses PDGF-BB-Induced HSCs Activation by Inhibiting the PDGF-BB/PDGFR-β Signaling Pathway

**Figure 7:**
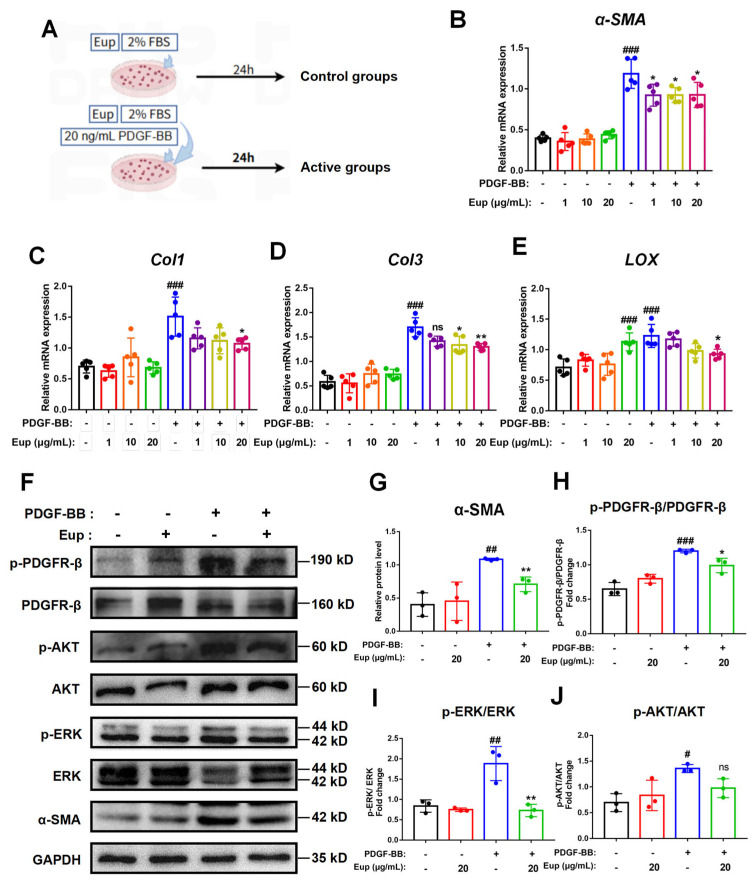
Eup suppresses PDGF-BB-induced HSC activation by inhibiting the PDGF-BB/PDGFR-β signaling pathway. (**A**) LX-2 cells were cultured with 20 ng/mL PDGF-BB, 1, 10, and 20 μg/mL Eup for 24 h. (**B**–**E**) qRT-PCR analysis of *α-SMA*, *Col1*, *Col3*, and *LOX* in LX-2 cells administrated with different concentrations of Eup. (**F**) Representative immunoblot images demonstrating protein expression of GAPDH, α-SMA, p-PDGFR-β, PDGFR-β, p-AKT, AKT, p-ERK, and ERK. (**G**–**J**) Expression levels of α-SMA and phosphorylation ratios of PDGFR-β (p-PDGFR-β/PDGFR-β), AKT (p-AKT/AKT), and ERK (p-ERK/ERK). The loading control was GAPDH. Data are presented as mean ± SD, n = 3. ^#^ *p* < 0.05, ^##^ *p* < 0.01, and ^###^ *p* < 0.001 versus PDGF-BB (-) Eup (-) group; * *p* < 0.05 and ** *p* < 0.01 versus PDGF-BB (+) Eup (-) group.

*In vitro*, we utilized PDGF-BB to activate LX-2 cells to further validate the anti-fibrotic mechanism of Eup (Figure 7A). Following the administration of PDGF-BB (20 ng/mL), qRT-PCR results indicated that the mRNA expression levels of *Col1*, *Col3*, *α-SMA*, and *LOX* were increased significantly. However, co-treatment with Eup (1, 10, and 20 μg/mL) for 24 h during the PDGF-BB-induced LX-2 cells activation, we observed that Eup (20 μg/mL) treatment significantly reduced the mRNA expression levels of *Col1*, *Col3*, *α-SMA*, and *LOX* in LX-2 cells (Figure 7B–E). Consistent with transcriptional changes, Western blotting results showed that PDGF-BB treatment significantly elevated the protein expression level of α-SMA in LX-2 cells, whereas Eup (20 μg/mL) treatment significantly reversed this effect (Figure 7F,G). Therefore, Eup can inhibit the activation of HSCs induced by PDGF-BB.

To further elucidate the mechanism by which Eup suppresses HSC activation, we investigated its potential regulatory effects on the PDGF-BB/PDGFR-β signaling cascade through Western blot analysis of key pathway components. As demonstrated in Figure 7F,H–J, stimulation of LX-2 cells with 20 ng/mL PDGF-BB resulted in significant elevation of phosphorylation ratios for PDGFR-β, AKT, and ERK. In contrast, Eup treatment (20 μg/mL) significantly attenuated these phosphorylation events. These results demonstrate that Eup effectively suppresses PDGF-BB-induced HSC activation through inhibition of the PDGF-BB/PDGFR-β signaling cascade. Collectively, the data indicate that Eup exerts its anti-fibrotic effects by targeting this pathway to inhibit HSC activation.

## 3. Discussion

In the United States, an estimated 80.19 million individuals are afflicted with Steatotic Liver Disease, with 14.32 million exhibiting Clinically Significant Fibrosis (CSF) [37]. In China, hepatic steatosis and associated fibrosis constitute a substantial health burden [38]. Unfortunately, there remains a paucity of clinically effective pharmacotherapies for liver fibrosis. Consequently, the development of efficacious interventions is imperative. Current therapeutic strategies for liver fibrosis primarily focus on: (1) inhibition of HSC activation, (2) induction of apoptosis in activated HSCs, (3) reduction of oxidative stress, (4) immune modulation, and (5) inhibition of ECM deposition and scar formation [39]. Given the multifactorial etiology of liver fibrosis, combination therapies targeting multiple pathways simultaneously are emerging as a key direction for future research [10]. In this context, traditional Chinese medicine (TCM) offers significant promise due to its multi-target, multi-pathway mechanisms of action, low toxicity, and favorable therapeutic outcomes [40,41]. Some Chinese traditional medicines have been shown to have the potential to improve liver fibrosis, such as Paeoniflorin [42], Notoginsenoside R1 [43], and Berberine [44]. Eup, identified in the 1970s, is predominantly utilized for the treatment of respiratory disorders. Empirical evidence suggests that Eup mitigates lung fibrosis and acute lung injury in murine models, and exerts inhibitory effects on the metastatic potential of hepatic and breast carcinomas [29,45,46]. This study aims to demonstrate that Eup may alleviate liver fibrosis caused by CCl_4_ administration in a murine model.

Through UPLC-Q/TOF-LC/MS, over one hundred components were identified in Eup. Among them, betaine has been shown to ameliorate dimethylnitrosamine (DMN)-induced oxidative liver injury by modulating sulfur-containing metabolite pathways, thereby attenuating oxidative stress and suppressing the progression of liver fibrosis [47]. Cyanidin is the aglycone moiety of Cyanidin-3-O-β-glucoside. The study demonstrates that Cyanidin-3-O-β-glucoside mitigates oxidative stress, reduces hepatocyte apoptosis, and suppresses hepatic inflammation, ultimately inhibiting HSC activation and preventing CCl_4_-induced liver fibrosis [48]. Furthermore, delphinidin has been reported to reverse progressive hepatic fibrosis by inactivating HSCs. This is achieved through the suppression of pro-fibrotic cytokines like TNF-α and TGF-β, alongside the upregulation of metallothionein I/II (MT I/II) to enhance hepatic regenerative capacity [49]. Our study systematically confirmed the anti-fibrotic properties of Eup using both *in vivo* and *in vitro* experimental approaches. *In vivo*, Eup administration significantly improved liver histopathology, as evidenced by reduced collagen fiber formation and ECM deposition. *In vitro*, Eup dramatically prevented the activation of LX-2 cells. Additionally, we noted that treatment with Eup alone did not affect normal liver function in mice, preliminarily indicating the safety of Eup.

Chronic liver inflammation is a primary driver in triggering and advancing the development of liver fibrosis [50]. Studies have shown that numerous pathways associated with inflammation are activated during the process of liver fibrosis, which promotes the activation of HSCs and the deposition of collagen in the liver [51]. Given the important role of inflammation in fibrosis, we verified whether Eup could exert an anti-inflammatory effect in CCl_4_-induced liver fibrosis. The former study has shown that IL-1β and TNF-α promote the survival of HSCs by mediating the activation of nuclear factor kappa B (NF-κB) within HSCs [52]. The level of plasma IL-6 may increase with the progression of cirrhosis [53]. Both resident hepatic macrophages and monocyte-derived macrophages are significant drivers of fibrosis progression. Our results indicated that Eup not only reduced the protein levels of pro-inflammatory cytokines in fibrotic livers, but also decreased the infiltration of neutrophils and monocytes. Macrophages, which play a central role in liver inflammation, can be polarized into two distinct phenotypes: M1 and M2. M1-phenotype macrophages, characterized by high expression of CD80/CD86 and inducible nitric oxide synthase (iNOS), exert pro-inflammatory effects, whereas M2-phenotype macrophages, marked by high expression of CD163/CD206, exhibit anti-inflammatory and tissue-repair functions [54]. Previous studies have shown that suppressing M1-phenotype macrophage polarization and stimulating M2-phenotype macrophage polarization alleviated mice liver fibrosis induced by CCl_4_ [55,56]. In alignment with these findings, our research revealed that Eup treatment increased the number of M2-phenotype macrophages while decreasing the number of M1-phenotype macrophages in the fibrotic livers of mice induced by CCl_4_. Therefore, the data suggest that Eup alleviates hepatic inflammatory responses by improving inflammatory cell infiltration, reducing the production of pro-inflammatory cytokines, and increasing the number of M2-phenotype macrophages, which contributes to the amelioration of liver fibrosis.

Using GSEA, we observed that treatment with Eup alone downregulated the PDGF/PDGFR-β signaling pathway in normal mouse liver. Over the past few years, PDGFR has emerged as a widely recognized target for anti-fibrotic therapeutics [20,21,22]. The level of PDGFR-β in liver tissue shows a positive correlation with the severity of liver fibrosis, and it is primarily expressed in activated HSCs [14]. Activation of PDGFR-β exacerbates liver fibrosis, as evidenced by increased collagen deposition and elevated mRNA expression of α-SMA and collagen I in mice with constitutive PDGFR-β activation [18]. PDGF-BB, a member of the PDGF family, is a ligand for PDGFR-β and also the most potent mitogen for HSCs. The expression of PDGF-BB is low in normal liver tissue, and the secretion of PDGF-BB is increased from hepatocytes and thrombocytes [57]. Here, we found that HSCs were remarkably activated after administering PDGF-BB. However, treatment with Eup significantly suppressed HSC activation induced by PDGF-BB. When PDGF-BB binds to the PDGFR-β on HSCs, tyrosine residues of PDGFR-β are phosphorylated, activating downstream signaling pathways including MAPK, PI3K/AKT, and Ras protein/tyrosine-protein kinase pathways [15,17,58]. Activation of the ERK and PI3K/AKT pathways supports the proliferation of HSCs and their differentiation into hepatic fibroblasts [59]. Similarly, stimulation of the p38 MAP kinase pathway enhances the migration of hepatic myofibroblasts [58]. In the current study, to further verify whether Eup improved CCl_4_-induced murine liver fibrosis by regulating the PDGF/PDGFR-β signaling pathway, we measured the phosphorylation levels of PDGFR-β, AKT, and ERK proteins. The findings indicated that Eup markedly decreased the phosphorylation levels of PDGFR-β, AKT, and ERK proteins both *in vivo* and *in vitro*. Collectively, we have confirmed Eup suppressed PDGF/PDGFR-β signaling pathway and its downstream key signaling proteins. Therefore, we conclude that Eup inhibits the activation of HSCs at least in part by regulating the PDGF/PDGFR-β signaling pathway, thereby improving CCl_4_-induced liver fibrosis in mice.

However, several limitations should be noted. First, Eup did not significantly ameliorate CCl_4_-induced liver injury, indicating that its primary therapeutic effects may be specific to fibrotic processes rather than acute hepatocyte damage. These results are consistent with existing studies [60,61]. This dissociation suggests that Eup’s primary action may be the selective modulation of HSCs activation, rather than direct protection of hepatocytes. Second, while betaine, cyanidin, and delphinidin have been identified as anti-fibrotic components, the contributions of other bioactive constituents in Eup remain to be elucidated. These bioactive components may exert their anti-fibrotic effects through multi-target mechanisms. Third, the mechanism by which Eup alleviates inflammation requires further investigation. Fourth, Eup treatment downregulated the protein expression of α-SMA, but failed to reduce the transcriptional level. The reason might be that Eup only functions during the translation process of α-SMA. Future studies should focus on isolating and characterizing additional active compounds in Eup, as well as exploring its molecular targets in greater depth to fully understand its therapeutic potential.

**Figure 8:**
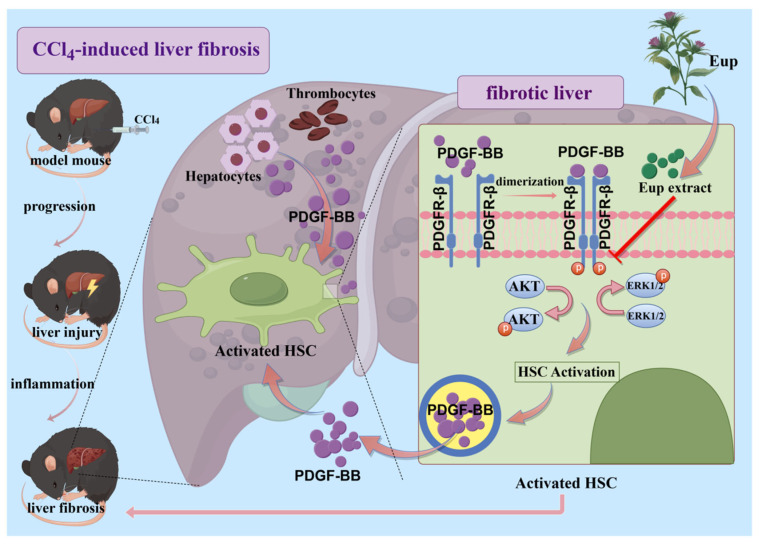
The mechanism diagram of Eup in improving CCl_4_-induced liver fibrosis. Eup suppressed the PDGF-BB/PDGFR-β signaling pathway, thereby inhibiting HSCs activation, and then improving liver fibrosis induced by CCl_4_.

In summary, our study demonstrates that Eup significantly ameliorates CCl_4_-induced liver fibrosis through dual mechanisms. As displayed in Figure 8, firstly, Eup inhibits the PDGF-BB/PDGFR-β signaling pathway, which subsequently suppresses the activation of HSCs and improves liver fibrosis. Secondly, Eup attenuates the inflammatory response in fibrotic livers, thereby mitigating the progression of liver fibrosis. The findings may provide potential therapeutic drugs for the clinical treatment of liver fibrosis and offer theoretical evidence for the development of clinical drugs for the treatment of liver fibrosis.

## 4. Materials and Methods

### 4.1. Preparation of Eup

Eup was purchased from Guangsheng Trading Co., Ltd. (Baoding, China), as a dried mixture of stems and leaves. Before decoction, the stems of Eup were rod-shaped, approximately 2 cm in length and 0.3 cm in diameter, while the leaf area was about 2 cm^2^. Eup was first soaked in 15 volumes of distilled water for 30 min, and a decoction was made twice for 1.5 h each time at 100 °C. Combine 2 times of water decoction and filter through sterile gauze, then concentrate to Eup containing 5 g crude drug per 1 mL under reduced pressure. The concentrated extract was aliquoted, sealed, and stored at −80 °C. For *in vitro* experiments, the extract was additionally filtered through a 0.22 µm sterile microporous membrane. The quantitative analysis of the primary constituents in Eup was conducted employing UPLC-Q/TOF-LC/MS. Liquid chromatography (LC) was performed using an Agilent 1290 UPLC system, and mass spectrometry (MS) was conducted using an Agilent Q-TOF 6550 instrument. The chromatographic method involved the use of water containing 0.1% formic acid as mobile phase A and acetonitrile as mobile phase B. The gradient elution program is presented in Table 3. The electrospray ionization parameters were optimized as follows: 4000 V for positive ion mode and 3200 V for negative ion mode. The sample injection volume was set at 5 µL.

### 4.2. Animals and Experimental Design

C57BL male mice (7 weeks; 18–22 g) were sourced from Zolgene Biotechnology Co., Ltd. (Nanjing, China). The housing environment for all mice was carefully controlled, maintaining a temperature of (24 ± 2) °C, relative humidity of (50 ± 5)%, and a pathogen-free condition, with the provision of sufficient food and water ad libitum.

Mice were deeply anesthetized via intraperitoneal injection of tribromoethanol, ensuring complete loss of consciousness and absence of pain perception. Then, euthanasia was immediately performed by cervical dislocation. Immediately post-mortem, dissection was performed. The target liver tissue was rapidly and aseptically excised. The excised liver tissue was promptly immersed in pre-chilled sterile saline or PBS buffer for a brief rinse to remove surface blood. Subsequently, the liver tissue was sectioned into small fragments (<0.5 cm^3^) to facilitate rapid freezing and subsequent homogenization.

The dissected liver fragments were immediately subjected to snap-freezing in liquid nitrogen. Following snap-freezing, the tissue samples were transferred to and stored long-term at −80℃ in an ultra-low temperature freezer until subsequent experimental analysis. All experimental procedures were conducted according to the guidelines approved by the Laboratory Animal Research Center of Fujian Medical University (ethical code: IACUC FJMU 2023-Y-1118, 23 November 2023).

A cohort of 30 mice was utilized in this study. Using GraphPad Prism software (version 9.0), animals underwent complete randomization and were allocated equally into six experimental groups (n = 5 each group): (1) control group, (2) 40 g/kg Eup group, (3) CCl_4_ group, (4) CCl_4_ + 4 g/kg Eup group, (5) CCl_4_ + 16 g/kg Eup group, (6) CCl_4_ + 40 g/kg Eup group), each group containing 5 mice. To induce liver fibrosis, CCl_4_ (2:5 *v*/*v* in corn oil) or an equal volume of corn oil was injected intraperitoneally (i.p.) at a dose of 2 mL/kg body weight (BW) once every two days during the experimental period (5 weeks). Concurrently, each group of mice was administered intragastrically (i.g.) with Eup (4, 16, and 40 g/kg) or an equal volume of a vehicle every day throughout the experimental period. Before sacrifice, the mice underwent a 12-h fasting period. Liver tissues and blood were collected for subsequent analyses. The experimental design is illustrated schematically in Figure 2A.

### 4.3. Histological Analysis: H&E Staining, Sirius-Red Staining, and Fibrotic Score

Liver tissues were immersed in 10% phosphate-buffered formalin for fixation, subsequently embedded in paraffin, and sectioned into 4 µm slices for histological examination.

To evaluate collagen deposition, liver tissue sections were stained with Sirius Red (G1472, Beijing Solarbio Science & Technology Co., Ltd., Beijing, China), using a saturated aqueous solution of picric acid combined with Direct Red 80. This staining technique specifically highlights the collagen fibers within the liver tissue, enabling a quantitative analysis of the fibrosis present. The quantitative analysis of the Sirius-red positive areas was executed via ImageJ software (Version 1.54f, National Institutes of Health, Bethesda, MD, USA).

Fibrotic changes were assessed through H&E staining, which was performed on paraffin-embedded tissue sections following standard protocols. The histological scoring system established by Ishak et al. was used to evaluate the stage of liver fibrosis [62].

### 4.4. Immunohistochemistry (IHC) Staining

As described before [63], liver tissues were fixed in 10% phosphate-buffered formalin, subsequently embedded in paraffin, and subjected to standard procedures. Using a microtome, tissue sections with a thickness of 4 µm were cut and subsequently mounted onto glass slides. Before staining, paraffin sections were deparaffinized by immersion in fresh xylene twice for 15 min each, rehydrated (successively immersed in 100%-90%-70%-50% ethanol for 2 min), and immersed in antigen repair solution (citrate) at 100 °C for 30 min. Then, blocking the Non-specific binding was carried out with 3% peroxidase solution at room temperature for 10 min. Afterward, the sections were incubated at 4 °C overnight with the respective primary antibodies. The primary antibodies used for immunohistochemistry included anti-CD86 (1:200), anti-CD163 (1:500), and anti-MPO (1:300). The sections were then incubated with horseradish peroxidase (HRP)-conjugated secondary antibodies at 37 °C for 30 min. Following each incubation step, the sections were rinsed three times with phosphate-buffered saline (PBS). The immune complexes were observed by using the DAB Substrate Kit according to the instructions from the manufacturer. Ultimately, the tissues were counterstained with hematoxylin and mounted for visualization under a light microscope. Image analysis was conducted using ImageJ software.

### 4.5. Serum Alanine Aminotransferase (ALT), Aspartate Aminotransferase (AST), and Alkaline Phosphatase (ALP) Detection

To evaluate hepatic damage, serum levels of ALT, AST, and ALP were measured. After collecting blood through cardiac puncture, the blood was first deposited at room temperature for 30 min, and then centrifuged at 3000 rpm for 15 min at 4 °C to isolate serum. The enzymatic activities of ALT, AST, and ALP were determined utilizing assay kits obtained from a commercial source (Nanjing Jiancheng Bioengineering Institute, Nanjing, China), adhering strictly to the protocols provided by the manufacturer.

### 4.6. Quantitative Real-Time Polymerase Chain Reaction (qRT-PCR)

Total RNA was isolated from both hepatic tissue and LX-2 cells employing the TRIzol reagent method (Takara, Japan), and treated with diethylpyrocarbonate (DEPC, BBI Life Sciences, Shanghai, China) to eliminate contaminants. The RNA purity was assessed by determining the absorbance ratio at 260/280 nm with a spectrophotometer (NanoDrop™, Thermo Fisher Scientific, Waltham, MA, USA). Subsequently, residual DNA was degraded using a 5x g-DNA digester Mix (Yeasen Biotechnology Co., Ltd., Shanghai, China) according to the manufacturer’s protocol. 1 µg RNA was reverse transcribed into cDNA using 4x Hifair^®^ III SuperMix Plus (Yeasen Biotechnology Co., Ltd., Shanghai, China). Quantitative PCR (qPCR) was conducted using Hieff^®^ qPCR SYBR Green Master Mix (Fujian Herui Biotechnology Co., Ltd., Fuzhou, China) on a CFX Connect™ Real-Time System (Bio-Rad Laboratories, Hercules, CA, USA). The identity of the amplified target cDNA was verified through melting curve analysis. Quantification of relative mRNA expression levels was standardized against glyceraldehyde-3-phosphate dehydrogenase (GAPDH), employing the 2^−ΔΔCt^ calculation approach. Detailed information regarding primer sequences is provided in Table 4.

### 4.7. Quantification of Inflammatory Cytokines by Enzyme-Linked Immunosorbent Assay (ELISA)

The protein levels of inflammatory cytokines (TNF-α, IL-1β, and IL-6) in liver tissue homogenates were quantified using commercial ELISA kits (Thermo Fisher Scientific, Waltham, MA, USA) following the manufacturer’s instructions.

### 4.8. Western Blot

Protein extraction from both CCl_4_-treated murine hepatic tissues and *in vitro* cultured cells was performed with Radio Immunoprecipitation Assay (RIPA) lysis buffer containing 1% protease and phosphatase inhibitor cocktail. The samples were kept on ice during the extraction process. After centrifugation at 12,000 rpm for 15 min at 4 °C, protein concentrations in the resulting supernatants were determined using a BCA protein quantification kit (HERUI) under the manufacturer’s guidelines. For Western blot analysis, primary antibodies against β-actin (1:2500, abs171598, Absin, Shanghai, China), α-SMA (1:2500, Proteintech Group, Wuhan, China), Collagen I (1:2000, Proteintech Group, Wuhan, China), PDGFR-β (1:1250, Proteintech Group, Wuhan, China), p-PDGFR-β (1:1000, Abcam, UK), GAPDH (1:1500, Servicebio, Wuhan, China), p-AKT (1:1000, Abcam, UK), AKT (1:1000, Abcam, UK), p-ERK (1:1500, Selleck Chemicals, Houston, TX, USA) and ERK (1:1000, Selleck Chemicals, Houston, TX, USA), were applied. Tris-buffered saline (TBS) was prepared by dissolving TBS powder (Biosharp) in double-distilled water. Protein samples (31.25 µg per lane) were subsequently separated by sodium dodecyl sulfate-polyacrylamide gel electrophoresis (SDS-PAGE) and subsequently transferred electrophoretically to polyvinylidene difluoride (PVDF) membranes. Subsequently, 5% non-fat milk or 5% bovine serum albumin (BSA), prepared in Tris-buffered saline with 0.1% Tween-20 (TBS-T), was used to incubate the membranes at room temperature for 1.5 h to achieve blocking. Afterward, the membranes were subjected to overnight incubation with specific primary antibodies at 4 °C (16–24 h), followed by a 1h incubation period at room temperature with corresponding secondary antibodies (Bioss, Beijing, China) at a dilution of 1:3000. After blocking and incubation with the primary or secondary antibodies, TBS-T was applied to wash the PVDF membranes. An enhanced chemiluminescence (ECL) detection system (Life-iLab, Shanghai, China) was employed to visualize the protein bands, and subsequent quantification of band intensity was performed utilizing ImageJ analysis software.

### 4.9. Cell Treatments

The LX-2 cells were obtained from Procell Life Science & Technology Co., Ltd. (Wuhan, China). The cultivation of LX-2 cells is conducted under controlled conditions at a temperature of 37 °C in an atmosphere enriched with 5% CO_2_. The cells are nurtured in Dulbecco’s Modified Eagle Medium (DMEM, BBI), which is further supplemented with 2% FBS (Nanjing SenBeiJia Biological Technology Co., Ltd., Nanjing, China) and 1% Penicillin-Streptomycin Solution to support their growth and to prevent microbial contamination. Adherent cell growth is monitored daily, and fresh medium is replaced to eliminate necrotic cells. Cells are passaged or digested for subsequent experiments when they reach 80–90% confluence. Cells in the logarithmic growth phase are used for experimentation to ensure the accuracy and reliability of experimental outcomes. To model fibrotic activation, LX-2 cells were stimulated with either (1) 20 ng/mL recombinant human PDGF-BB (TargetMol, MA, USA) for 24 h, or (2) 10% FBS-containing DMEM for 24 h. Control groups received equivalent volumes of serum-free DMEM with vehicle (PBS).

### 4.10. RNA-Sequencing Analysis

Liver tissues were rapidly excised, homogenized in TRIzol, and snap-frozen in liquid nitrogen. RNA sequencing was performed by Wuhan Kangce Biotechnology Co., Ltd. (Wuhan, China). Sequencing analysis was performed on the Illumina NovaSeq 6000 platform (Illumina, CA, USA). For differential gene expression analysis, the DESeq2 package was implemented. Identification of differentially expressed genes (DEGs) was based on the established criteria of |fold change| ≥ 2 and a false discovery rate (FDR) threshold of < 0.05.

Functional profiling of DEGs was conducted via GSEA and KEGG pathway enrichment to uncover significantly modulated biological processes and pathway signatures. Transcriptomic profile variations between normal control and Eup-treated groups were analyzed through hierarchical clustering and principal component analysis (PCA) approaches. The R package heatmap (R4.4.1) was utilized to generate a graphical representation of DEGs.

### 4.11. Statistical Analysis

GraphPad Prism software (Version 9.0.0, San Diego, CA, USA) was applied for statistical analysis and graphing column charts with individual values. All experimental results are expressed as mean values with standard deviation (mean ± SD). Before statistical comparisons, datasets were subjected to normality assessment using the Shapiro-Wilk test and variance homogeneity verification through Bartlett’s test. Statistical analysis of normally distributed datasets with homogeneous variances across groups (*p* > 0.05) was performed using one-way analysis of variance (ANOVA) with subsequent Tukey’s post hoc testing for multiple comparison analysis. When variance homogeneity assumptions were violated (*p* < 0.05), non-parametric alternatives, including the Brown-Forsythe test for variance homogeneity and Welch’s ANOVA with Games-Howell post hoc analysis, were employed. Statistical significance was determined at a threshold of *p* < 0.05. The statistical significances of different grades were defined as * *p* < 0.05, ** *p* < 0.01, and *** *p* < 0.001.

## 5. Conclusions

Eup improves CCl_4_-induced liver fibrosis by alleviating hepatic inflammation and suppressing the HSCs activation via modulating the PDGF-BB/PDGFR-β signaling pathway.

## Figures and Tables

**Table 1:** Components of Eup identified by UPLC-Q/TOF-LC/MS in negative ion mode.

NO.	Retention Time (min)	Molecular Formula	(*m*/*z*) (Mass Error) (ppm)	Identification
1	0.344	C_6_H_8_O_7_	192.027 (−2.55)	Citric acid
2	1.107	C_4_H_6_O_6_	150.0164 (−0.57)	Tartaric acid
3	1.107	C_9_H_9_NO_4_	195.0532 (−0.88)	Peristrophamide
4	1.107	C_8_H_7_NO_3_	165.0426 (−5.22)	Coixol
5	1.202	C_5_H_10_O_5_	150.0528 (−8.36)	Apiose
6	1.202	C_17_H_21_N_4_O_9_P	456.1046 (−0.49)	Flavin mononucleotide
7	1.297	C_4_H_6_O_5_	134.0215 (−2.59)	Malic acid
8	1.393	C_25_H_28_O_11_	504.1632 (−2.63)	Shakuchirin
9	1.488	C_3_H_6_O_3_	90.0317 (−7.45)	Dihydroxyacetone
10	1.583	C_5_H_4_O_3_	112.016 (−0.53)	Pyromeconic acid
11	1.583	C_6_H_8_O_7_	192.027 (−0.68)	Citric acid
12	1.869	C_24_H_42_O_21_	666.2219 (−1.13)	Isolychnose
13	1.869	C_6_H_6_O_3_	126.0317 (−6.48)	4-Hydroxymethyl-2-furaldehyde
14	2.155	C_4_H_6_O_2_	86.0368 (0.04)	Crotonic acid
15	2.155	C_5_H_6_O_4_	130.0266 (−1.72)	Mesaconic acid
16	2.346	C_30_H_52_O_26_	828.2747 (−1.76)	Verbascose
17	2.441	C_6_H_8_O_7_	192.027 (0.33)	Citric acid
18	2.441	C_5_H_4_O_3_	112.016 (−3.93)	Pyromeconic acid
19	2.441	C_6_H_10_O_5_	162.0528 (−3.47)	3,6-Anhydrogalactose
20	2.727	C_7_H_12_O_6_	192.0634 (6.06)	Cordycepic acid
21	3.013	C_7_H_6_O_5_	170.0215 (1.32)	Gallic acid
22	3.013	C_12_H_17_N_3_O_8_	331.1016 (0.96)	Tetrodonic acid
23	3.395	C_6_H_8_O_7_	192.027 (−0.67)	Citric acid
24	3.490	C_7_H_14_O_4_	162.0892 (0.91)	Cymarose
25	3.776	C_7_H_6_O_3_	138.0317 (−1.85)	3,4-Dihydroxybenzyl aldehyde
26	3.871	C_6_H_6_O	94.0419 (−3.5)	Phenol
27	4.157	C_8_H_8_O_2_	136.0524 (−2.94)	4-Methyl salicylaldehyde
28	4.157	C_9_H_8_O_4_	180.0423 (−3.49)	Caffeic acid
29	4.253	C_6_H_10_O_8_	210.0376 (0.13)	Mucic acid
30	4.539	C_7_H_6_O_4_	154.0266 (−4.48)	3,5-Dihydroxybenzoic acid
31	4.825	C_25_H_24_O_12_	516.1268 (−2.14)	1,4-Dicaffeoylquinic acid
32	4.920	C_21_H_20_O_11_	448.1006 (−3.68)	3,3’4’,5,7- Pentahydroxyvone-3-L-rhamnoside
33	5.015	C_11_H_20_O_5_	232.1311 (−4.49)	Jioglutin E
34	5.015	C_2_H_2_O_4_	89.9953 (−6.64)	Oxalic acid
35	5.015	C_15_H_10_O_6_	286.0477 (0.29)	5,7,2’,3’- Tetrahydroxyflavone
36	5.015	C_9_H_8_O_5_	196.0372 (−1.59)	Meconic acid
37	5.397	C_20_H_26_O_6_	362.1729 (0.84)	Tetrahydroxy-ent-Kaur-16-en-6,15-dione
38	5.492	C_16_H_12_O_7_	316.0583 (0.59)	3-Methoxy quercetin
39	5.873	C_5_H_10_O_2_	102.0681 (−3.97)	2-Methyl butyric acid
40	5.873	C_18_H_34_O_5_	330.2406 (−3.27)	Sanleng acid
41	5.873	C_15_H_10_O_6_	286.0477 (−4.67)	5,7,2’,3’-Tetrahydroxyflavone
42	5.873	C_16_H_12_O_7_	316.0583 (−3.52)	3-Methoxy quercetin
43	6.350	C_6_H_8_O_7_	192.027 (−0.98)	Citric acid
44	6.350	C_5_H_8_O_4_	132.0423 (−2.05)	2,3-Dihydroxyl-2-methyl-butyrolactone
45	6.350	C_20_H_24_O_6_	360.1573 (−1.2)	3-(alpha,4-Dihydroxy-3-methoxybenzyl)-4-(hydroxy-3-methoxybenzyl) tetrahydrofuran
46	6.922	C_17_H_26_O_4_	294.1831 (−3.36)	Embelin
47	6.922	C_13_H_16_O_4_	236.1049 (−0.75)	Asarumin B
48	6.922	C_12_H_19_N_3_O	221.1528 (−5.49)	Alchorneine
49	7.113	C_15_H_16_O_2_	228.115 (−6.07)	7-Hydroxycadalenal

**Table 2:** Components of Eup identified by UPLC-Q/TOF-LC/MS in positive ion mode.

NO.	Retention Time (min)	Molecular Formula	(*m*/*z*) (Mass Error) (ppm)	Identification
1	1.008	C_5_H_11_NO_3_	133.0739 (−1.51)	1,4-Dideoxy-1,4-imino-arabinitol
2	1.103	C_6_H_8_O_7_	192.0270 (−1.56)	Citric acid
3	1.199	C_5_H_11_NO_2_	117.0790 (−1.00)	Betaine
4	1.199	C_7_H_7_NO_2_	137.0477 (0.05)	Trigonelline
5	1.389	C_6_H_11_NO_2_	129.0790 (1.24)	6xi-Methoxypiperidin-2-one
6	1.484	C_8_H_13_NO_2_	155.0946 (−0.36)	Arecolidine
7	1.58	C_6_H_7_NO_2_	125.0477 (−2.7)	5-Hydroxy-2-pyridine methanol
8	1.58	C_9_H_15_NO_2_	169.1103 (−0.36)	Homoarecoline
9	1.58	C_8_H_13_NO_3_	171.0895 (0.9)	Desmodilactone
10	1.675	C_5_H_11_N_3_O_2_	145.0851 (−1.92)	gamma-Guanidinobutyric acid
11	1.675	C_8_H_15_NO	141.1154 (−1.35)	Hygrine
12	1.675	C_11_H_15_NO_7_	273.0849 (−1.13)	Brachystemoside A
13	1.961	C_5_H_7_NO_3_	129.0426 (−1.48)	Pyroglutamic acid
14	2.152	C_5_H_7_NO_2_	113.0477 (−2.05)	1-Cyano-2-hydroxymethyl prop-1-ene-3-ol
15	2.152	C_11_H_17_NO_7_	275.1005 (−2.05)	Cardiospermin
16	2.342	C_12_H_16_O_8_	288.0845 (−0.38)	Phlorin
17	2.628	C_12_H_17_NO_6_	271.1056 (0.84)	Deidaclin
18	2.628	C_9_H_17_NO_8_	267.0954 (5.33)	Miserotoxin
19	2.628	C_10_H_13_N_5_O_4_	267.0968 (0.65)	Adenosine
20	2.819	C_6_H_6_O_3_	126.0317 (2.56)	4-Hydroxymethyl-2-furaldehyde
21	3.105	C_7_H_8_O_4_	156.0423 (−1.09)	Doederleinic acid
22	3.296	C_8_H_7_NO	133.0528 (−2.28)	Mandelonitrile
23	3.296	C_16_H_27_NO_5_	313.1889 (−1.36)	Heliotrine
24	3.296	C_15_H_25_NO_5_	299.1733 (3.33)	Echinatine
25	3.296	C_15_H_27_NO_5_	301.1889 (−5.66)	Floridinine
26	3.391	C_8_H_5_NO_2_	147.0320 (−0.92)	Isatin
27	3.582	C_16_H_18_O_9_	354.0951 (0.88)	4-O-Caffeoyl-D-quinic acid
28	3.677	C_8_H_15_NO	141.1154 (−2.59)	Hygrine
29	3.772	C_8_H_8_O_2_	136.0524 (0.38)	4-Methyl salicylaldehyde
30	3.868	C_8_H_10_O_4_	170.0579 (−1.44)	Dictafolin B
31	3.963	C_16_H_24_O_10_	376.1370 (3.37)	6-O-Methyl catalpol
32	3.963	C_17_H_20_N_4_O_6_	376.1383 (0.04)	Vitamin B2
33	3.963	C_13_H_18_O_2_	206.1307 (−0.18)	Arteamisinine I
34	4.058	C_20_H_26_O_7_	378.1679 (−0.14)	1-(4-Hydroxy-3-methoxyphenyl-2-[4-(omega-hydroxypropyl)-2-methoxyphenoxy]propane-1,3-diol
35	4.058	C_17_H_27_NO_6_	341.1838 (−0.18)	Acetylindicine
36	4.154	C_21_H_26_O_10_	438.1526 (−0.76)	Bruceolide
37	4.154	C_26_H_28_O_14_	564.1479 (−1.35)	5,7,4’-Trihydroxy-6-C-arabinoside-8-C-glucoside flavone
38	4.249	C_17_H_20_O_9_	368.1107 (0.79)	Methyl chlorogenate
39	4.440	C_25_H_26_O_13_	534.1373 (−2.42)	6-beta-C-(2’-Galloylglucopyranosyl)-5,7-dihydroxy-2-isopropyl chromone
40	4.535	C_14_H_23_NO_6_	301.1525 (−1.49)	Intermediate
41	4.535	C_15_H_10_O_7_	302.0427 (−1.03)	3,5,7,2’,6’-Pentahydroxy flavonol
42	4.535	C_15_H_11_O_7_	303.0505 (−1.04)	Delphinidin
43	4.535	C_20_H_31_NO_8_	413.2050 (0.01)	Heliosupine N-oxide
44	4.726	C_15_H_11_O_6_	287.0556 (−1.06)	Cyanidin
45	4.726	C_15_H_10_O_6_	286.0477 (−1.06)	5,7,2’,3’-Tetrahydroxyflavone
46	4.726	C_21_H_20_O_12_	464.0955 (2.55)	6-Hydroxykaempferol-7-O-glucoside
47	4.726	C_27_H_31_O_15_	595.1663 (−1.20)	Pelargonidin-3,5-diglucoside
48	4.726	C_27_H_30_O_15_	594.1585 (−1.19)	6,8-Bis(C-glucosyl)-apigenin
49	4.821	C_21_H_20_O_11_	448.1006 (−0.72)	3,3’4’,5,7-Pentahydroxyvone-3-L-rhamnoside
50	4.821	C_21_H_21_O_11_	449.1084 (−0.54)	Cyanidin 3-O-beta-D-galactoside
51	5.012	C_15_H_24_O	220.1827 (−4.78)	(-)-1,10-Epoxy-guaia-11-ene
52	5.393	C_22_H_28_O_8_	420.1784 (−0.32)	Caesalmin A
53	5.393	C_13_H_12_O_2_	200.0837 (−1.29)	4,4’-Dihydroxydiphenyl methane
54	5.488	C_30_H_46_O_3_	454.3447 (−2.93)	(24Z)-27-Hydroxy-3-oxo-7,24-tirucalladien-21-al
55	5.488	C_15_H_16_O_2_	228.1150 (0.28)	7-Hydroxycadalenal
56	5.488	C_16_H_12_O_7_	316.0583 (−1.74)	3-Methoxy quercetin
57	5.488	C_14_H_14_O_2_	214.0994 (−4.68)	Lunularin
58	5.584	C_15_H_22_	202.1722 (−0.16)	1,2,9,10-Tetradehydroaristolane
59	5.584	C_15_H_24_O	220.1827 (−2.36)	(-)-1,10-Epoxy-guaia-11-ene
60	5.679	C_15_H_24_N_2_O	248.1889 (−1.14)	Aphylline
61	6.251	C_14_H_22_O_2_	222.1620 (−5.78)	[Z, E]-4,8,12-Trimethyl-3,7,11-tridecatrienoate
62	6.346	C_24_H_30_O_9_	462.1890 (−1.85)	1,1’-Dibenzene-6’,8’,9’-trihydroxy-3-allyl-4-O-beta-D-glucopyranoside
63	6.346	C_20_H_24_O_6_	360.1573 (−1.65)	3-(alpha,4-Dihydroxy-3-methoxybenzyl)-4-(hydroxy-3-methoxybenzyl) tetrahydrofuran
64	6.346	C_15_H_16_O_2_	228.1150 (−0.52)	7-Hydroxycadalenal
65	6.346	C_15_H_14_O	210.1045 (−1.36)	Linderazulene
66	6.346	C_17_H_25_NO_2_	275.1885 (−0.53)	Hydroxy-gamma-Sanshool
67	6.537	C_12_H_22_O	182.1671 (−1.91)	Cyclododecanone
68	6.537	C_13_H_20_O	192.1514 (−1.48)	beta-Ionone
69	6.537	C_21_H_27_NO_7_	405.1788 (−0.51)	Clivorine
70	6.632	C_17_H_26_O_4_	294.1831 (0.02)	Embelin
71	6.632	C_17_H_24_O_3_	276.1725 (−1.36)	6-Shogaol
72	7.014	C_17_H_26_O_5_	310.1780 (−1.27)	2-(1-Ethoxy-2-hydroxy)propyl-4-methoxyphenyl-2-methyl-butyrate
73	7.109	C_20_H_27_NO_5_	361.1889 (−2.09)	Cephalofortuneine
74	7.204	C_27_H_28_N_2_O_4_	444.2049 (−1.58)	Trichosanatine
75	7.204	C_21_H_24_O_6_	372.1573 (−1.97)	Fargesone A
76	7.204	C_21_H_34_O_10_	446.2152 (−0.09)	(Z)-(IS,5R)-beta-Pinen-10-yl-beta-vicianoside
77	7.3	C_9_H_10_	118.0783 (−0.20)	Isoallylbenzene
78	7.3	C_24_H_30_O_6_	414.2042 (−1.24)	Armilliaripin
79	7.3	C_19_H_18_O_4_	310.1205 (−1.45)	3alpha-Hydroxytanshinone IIA
80	7.49	C_14_H_22_	190.1722 (−3.27)	4-(1,5-Dimethyl-1,4-hexadienyl)-1-methyl-cyclohexene
81	8.539	C_17_H_30_O	250.2297 (−0.73)	Civetone
82	8.539	C_24_H_41_NO_7_	455.2883 (−0.91)	10-Hydroxynudicaulidine
83	8.539	C_18_H_33_NO_2_	295.2511 (−0.39)	Tetrahydrobungeanool
84	8.539	C_18_H_30_O_2_	278.2246 (−1.58)	(Z,Z,Z)-9,12,15-Octadecatrienoic acid
85	9.016	C_8_H_4_O_3_	148.0160 (−1.83)	Phthalic anhydride
86	10.446	C_35_H_42_O_12_	654.2676 (−0.88)	13-Deacetoxy-13,15-epoxy-11(15- > 1)-abeo-13-epi-baccatin VI
87	11.78	C_24_H_38_O_4_	390.2770 (−1.92)	3alpha-Hydroxy-6-oxo-5alpha-cholanic acid
88	14.736	C_6_H_15_N	101.1205 (−4.14)	Hexyl amine-1
89	17.786	C_5_H_5_NO_2_	111.0320 (−3.51)	2-Minaline

**Table 3:** Table of the column mobile phase gradient conditions.

Time (min)	Flow (mL/min)	Phase A (%)	Phase B (%)
0	0.3	97	3
3	0.3	75	25
4	0.3	55	45
10	0.3	5	95
13	0.3	5	95
17	0.3	97	3
20	0.3	97	3

**Table 4:** Primer sequences for real-time reverse transcription polymerase chain reaction.

Gene Symbol	Forward 5’-3’	Reverse 5’-3’
Mice		
*GAPDH*	AGGTCGGTGTGAACGGATTTG	GGGGTCGTTGATGGCAACA
*Col1*	GCTCCTCTTAGGGGCCACT	ATTGGGGACCCTTAGGCCAT
*Col3*	CTGTAACATGGAAACTGGGGAAA	CCATAGCTGAACTGAAAACCACC
*Col4*	CCTGGCACAAAAGGGACGA	ACGTGGCCGAGAATTTCACC
*LOX*	CAGCCACATAGATCGCATGGT	GCCGTATCCAGGTCGGTTC
*TGF-β*	CCACCTGCAAGACCATCGAC	CTGGCGAGCCTTAGTTTGGAC
*IL-10*	CTTACTGACTGGCATGAGGATCA	GCAGCTCTAGGAGCATGTGG
Human		
*GAPDH*	GGAGCGAGATCCCTCCAAAAT	GGCTGTTGTCATACTTCTCATGG
*Col1*	GAGGGCCAAGACGAAGACATC	CAGATCACGTCATCGCACAAC
*Col3*	GGAGCTGGCTACTTCTCGC	GGGAACATCCTCCTTCAACAG
*LOX*	CGGCGGAGGAAAACTGTCT	TCGGCTGGGTAAGAAATCTGA
*α-SMA*	AAAAGACAGCTACGTGGGTGA	GCCATGTTCTATCGGGTACTTC

## Data Availability

The original contributions presented in this study are included in the article/supplementary material. Further inquiries can be directed to the corresponding authors.

## Supplementary Materials

The following supporting information can be downloaded at: https://www.mdpi.com/article/10.3390/ph18081228/s1, Supplementary Figure S1: Food intake, initial body weight, final body weight, liver weight, and liver index of the mice in each group; Supplementary Figure S2: The analyses of GSEA; Supplementary Figure S3: Cluster heatmap. Supplementary Figure S4: Western blot bands.

## Abbreviations

Eup	*Eupatorium lindleyanum* DC
PCA	Principal Component Analysis
GSEA	Gene Set Enrichment Analysis
HSC	Hepatic stellate cells
CLD	Chronic liver disease
ECM	Extracellular matrix
ROS	Reactive oxygen species
TGF-β1	Transforming growth factor-beta 1
PDGFR	Platelet-Derived Growth Factor Receptor
BW	Body weight
i.g.	Intragastrically
i.p.	Intraperitoneally
H&E staining	Haematoxylin and eosin (H&E) staining
IHC	Immunohistochemistry staining
HRP	Horseradish peroxidase
ALT	Alanine aminotransferase
AST	Aspartate aminotransferase
GAPDH	Glyceraldehyde-3-phosphate dehydrogenase
RIPA	Radioimmunoprecipitation assay buffer
TBS	Tris-buffered saline
SDS-PAGE	Sodium dodecyl sulphatepolyacrylamide gel electrophoresis
PVDF	Polyvinylidene difluoride
BSA	Bovine serum albumin
ECL	Enhanced chemiluminescence
FBS	Fetal Bovine Serum
DEGs	Differentially expressed genes
FDR	False discovery rate
GO	Gene Ontology
KEGG	Kyoto Encyclopedia of Genes and Genomes
UPLC-Q/TOF-LC/MS	Ultra-performance liquid chromatography coupled with quadrupole time-of-flight mass spectrometry
α-SMA	α-smooth muscle actin
Col1	Collagen type I
Col3	Collagen type III
LOX	Lysyl oxidase
ERK	PI3K/AKT and MAPK pathways
CSF	Clinically Significant Fibrosis
TCM	Traditional Chinese medicine
DMN	Dimethylnitrosamine
NF-κB	Nuclear factor kappa B
iNOS	Inducible nitric oxide synthase
MAPK	Mitogen-activated protein kinase
