# Supplementary material for: Eupatorium lindleyanum DC Ameliorates Carbon Tetrachloride-Induced Hepatic Inflammation and Fibrotic Response in Mice
Source: Pharmaceuticals (Basel). 2025 Aug 20;18(8):1228. doi: 10.3390/ph18081228 (PMC12389491; doi:10.3390/ph18081228)
Supplement: Supplementary file 1 [file pharmaceuticals-18-01228-s001.zip › pharmaceuticals-3790117-supplementary.zip-2/Supplementary Figure 04.pptx]

## Slide 1
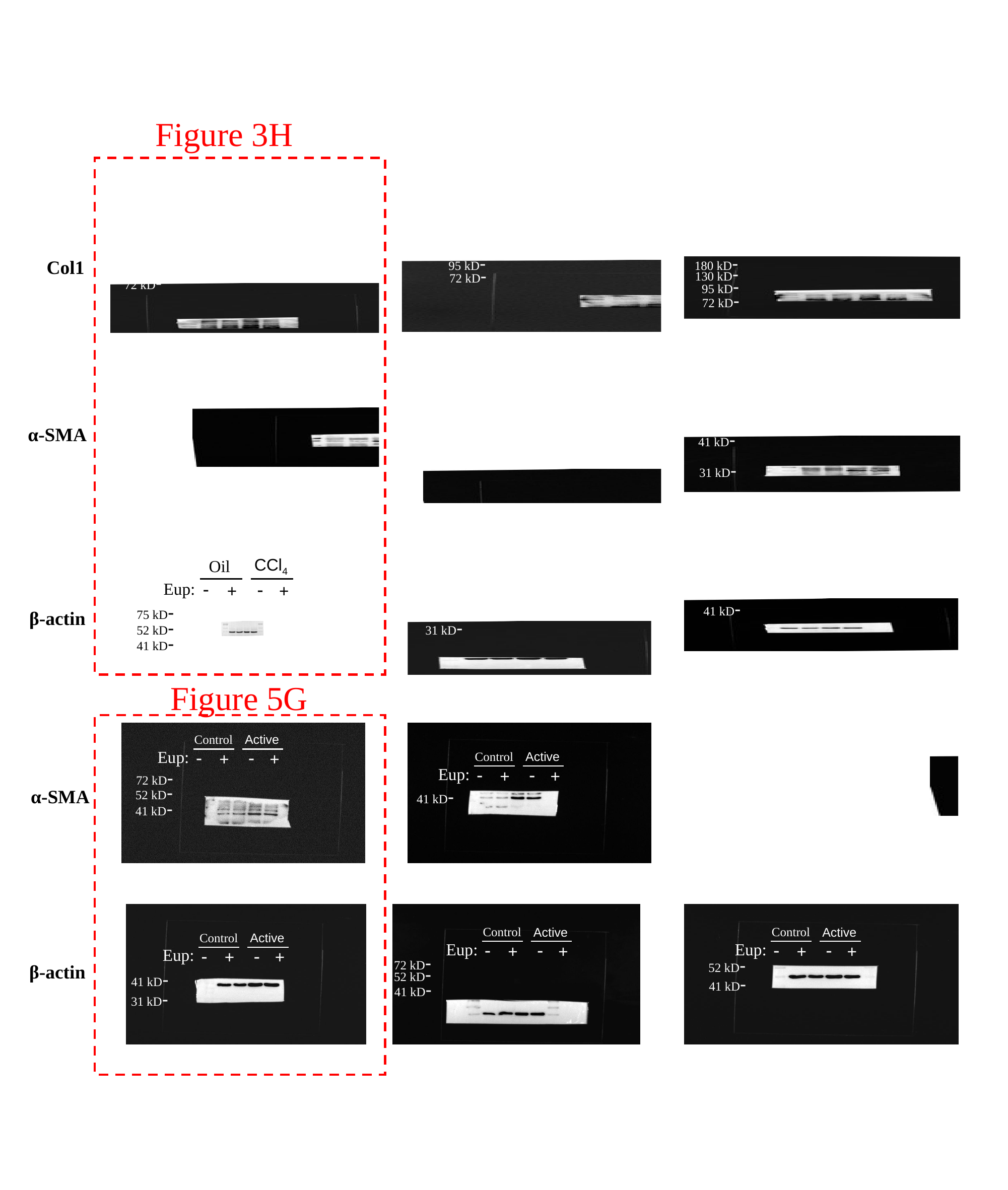

Oil
CCl4
-
-
+
+
Eup:
Figure 3H
180 kD-
130 kD-
95 kD-
72 kD-
Oil
CCl4
-
-
+
+
Eup:
180 kD-
130 kD-
95 kD-
72 kD-
Oil
CCl4
-
-
+
+
Eup:
Oil
CCl4
-
-
+
+
Eup:
Col1
Oil
CCl4
-
-
+
+
Eup:
Oil
CCl4
-
-
+
+
Eup:
41 kD-
52 kD-
31 kD-
Oil
CCl4
-
-
+
+
Eup:
52 kD-
41 kD-
31 kD-
52 kD-
41 kD-
31 kD-
α-SMA
Oil
CCl4
-
-
+
+
Eup:
52 kD-
41 kD-
Oil
CCl4
-
-
+
+
Eup:
41 kD-
31 kD-
CCl4
Oil
-
-
+
+
Eup:
75 kD-
52 kD-
41 kD-
β-actin
72 kD-
52 kD-
41 kD-
Control
Active
-
-
+
+
Eup:
Control
Active
-
-
+
+
Eup:
41 kD-
Control
Active
-
-
+
+
Eup:
72 kD-
52 kD-
41 kD-
α-SMA
Control
Active
-
-
+
+
Eup:
41 kD-
31 kD-
Control
Active
-
-
+
+
Eup:
72 kD-
52 kD-
41 kD-
Control
Active
-
-
+
+
Eup:
41 kD-
52 kD-
β-actin
130 kD-
180 kD-
95 kD-
72 kD-
Figure 5G
Oil
CCl4
-
-
+
+
Eup:

## Slide 2
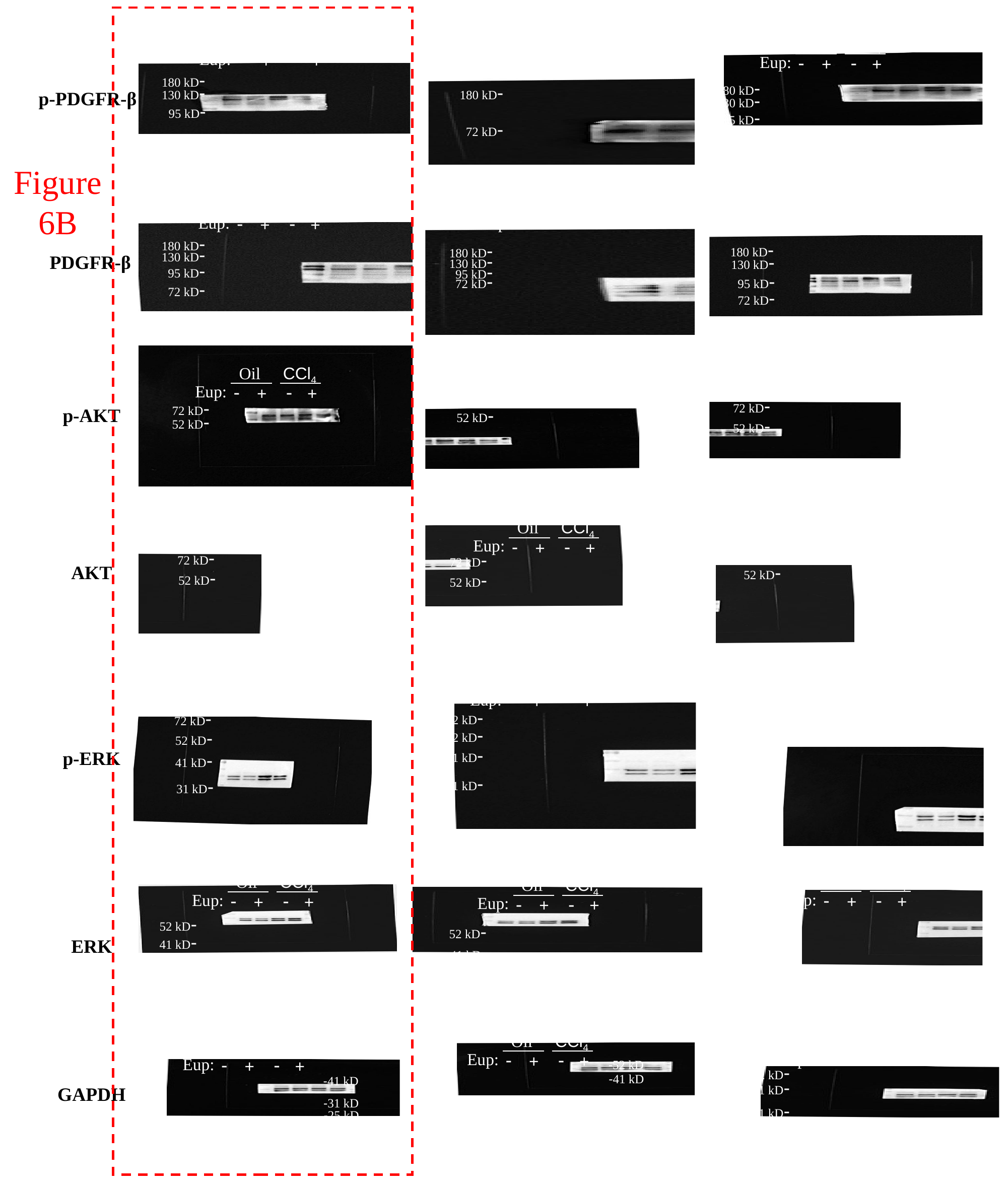

180 kD-
130 kD-
95 kD-
180 kD-
72 kD-
180 kD-
130 kD-
95 kD-
72 kD-
p-PDGFR-β
Oil
CCl4
-
-
+
+
Eup:
Oil
CCl4
-
-
+
+
Eup:
Oil
CCl4
-
-
+
+
Eup:
180 kD-
130 kD-
95 kD-
72 kD-
180 kD-
130 kD-
95 kD-
72 kD-
95 kD-
72 kD-
180 kD-
130 kD-
PDGFR-β
Oil
CCl4
-
-
+
+
Eup:
Oil
CCl4
-
-
+
+
Eup:
Oil
CCl4
-
-
+
+
Eup:
72 kD-
52 kD-
72 kD-
52 kD-
72 kD-
52 kD-
p-AKT
Oil
CCl4
-
-
+
+
Eup:
Oil
CCl4
-
-
+
+
Eup:
Oil
CCl4
-
-
+
+
Eup:
72 kD-
52 kD-
72 kD-
52 kD-
72 kD-
52 kD-
AKT
Oil
CCl4
-
-
+
+
Eup:
Oil
CCl4
-
-
+
+
Eup:
Oil
CCl4
-
-
+
+
Eup:
72 kD-
52 kD-
41 kD-
31 kD-
72 kD-
52 kD-
41 kD-
31 kD-
52 kD-
41 kD-
31 kD-
p-ERK
Oil
CCl4
-
-
+
+
Eup:
Oil
CCl4
-
-
+
+
Eup:
Oil
CCl4
-
-
+
+
Eup:
52 kD-
41 kD-
31 kD-
52 kD-
41 kD-
31 kD-
52 kD-
41 kD-
31 kD-
ERK
Oil
CCl4
-
-
+
+
Eup:
Oil
CCl4
-
-
+
+
Eup:
-41 kD
-31 kD
-25 kD
-41 kD
-31 kD
-25 kD
-52 kD
52 kD-
41 kD-
25 kD-
31 kD-
GAPDH
Oil
CCl4
-
-
+
+
Eup:
Oil
CCl4
-
-
+
+
Eup:
Oil
CCl4
-
-
+
+
Eup:
Oil
CCl4
-
-
+
+
Eup:
72 kD-
Figure 6B
Oil
CCl4
-
-
+
+
Eup:

## Slide 3
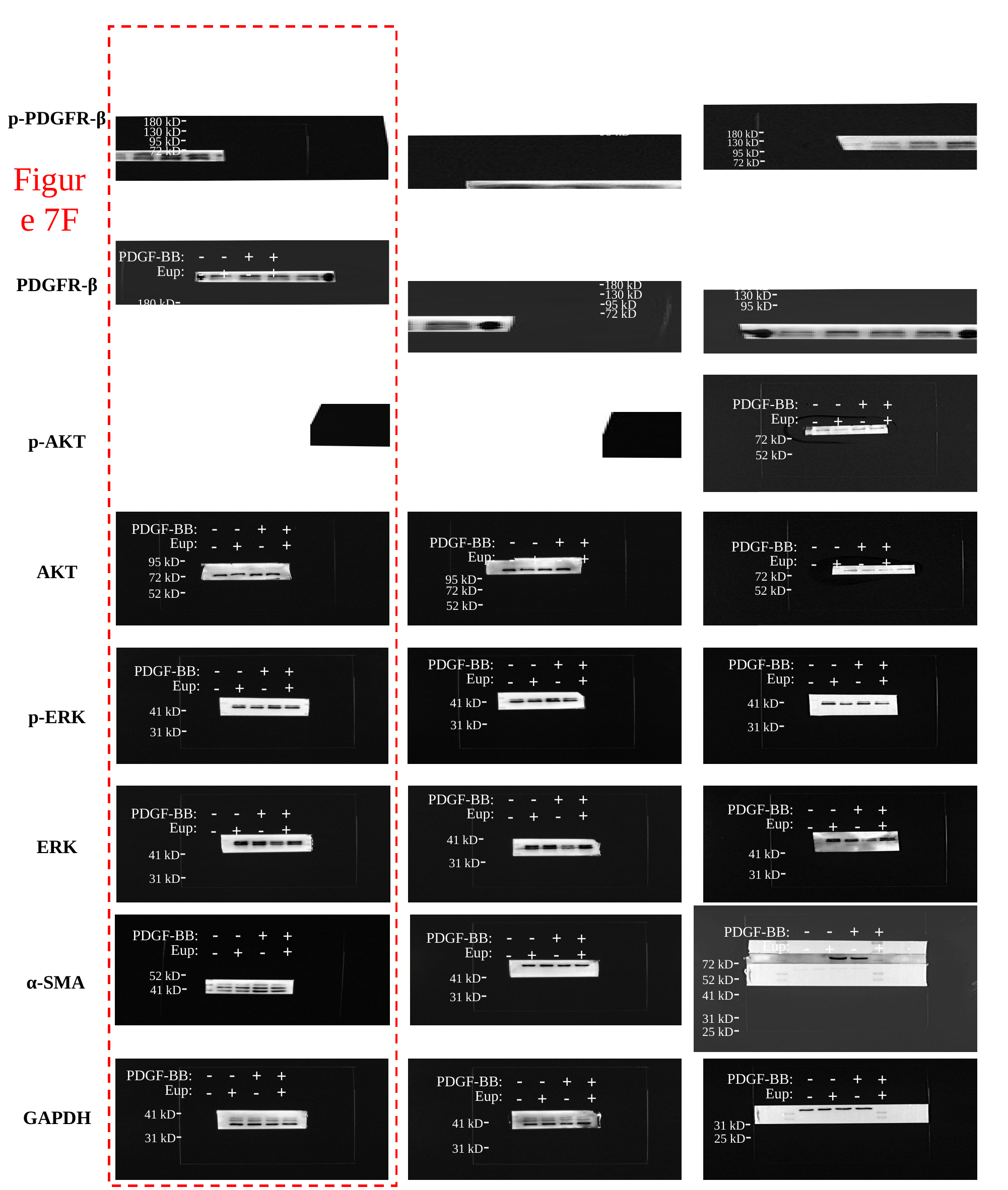

-
-
+
+
PDGF-BB:
-
-
+
+
Eup:
130 kD-
95 kD-
180 kD-
72 kD-
-
-
+
+
PDGF-BB:
-
Eup:
-
+
+
-180 kD
-95 kD
-
-
+
+
PDGF-BB:
-
Eup:
-
+
+
180 kD-
130 kD-
72 kD-
95 kD-
p-PDGFR-β
Figure 7F
180 kD-
-
-
+
+
PDGF-BB:
-
Eup:
-
+
+
95 kD-
130 kD-
-180 kD
-95 kD
-130 kD
-72 kD
-
-
+
+
PDGF-BB:
-
Eup:
-
+
+
180 kD-
130 kD-
95 kD-
-
-
+
+
PDGF-BB:
-
Eup:
-
+
+
PDGFR-β
-
-
+
+
PDGF-BB:
-
Eup:
-
+
+
95 kD-
72 kD-
52 kD-
95 kD-
72 kD-
52 kD-
-
-
+
+
PDGF-BB:
-
Eup:
-
+
+
52 kD-
72 kD-
-
-
+
+
PDGF-BB:
-
Eup:
-
+
+
p-AKT
72 kD-
-
-
+
+
PDGF-BB:
-
Eup:
-
+
+
95 kD-
52 kD-
-
-
+
+
PDGF-BB:
-
Eup:
-
+
+
95 kD-
72 kD-
52 kD-
-
-
+
+
PDGF-BB:
-
Eup:
-
+
+
52 kD-
72 kD-
AKT
-
-
+
+
PDGF-BB:
-
Eup:
-
+
+
41 kD-
31 kD-
-
-
+
+
PDGF-BB:
-
Eup:
-
+
+
31 kD-
41 kD-
-
-
+
+
PDGF-BB:
-
Eup:
-
+
+
41 kD-
31 kD-
p-ERK
-
-
+
+
PDGF-BB:
-
Eup:
-
+
+
41 kD-
31 kD-
-
-
+
+
PDGF-BB:
-
Eup:
-
+
+
41 kD-
31 kD-
-
-
+
+
PDGF-BB:
-
Eup:
-
+
+
41 kD-
31 kD-
ERK
-
-
+
+
PDGF-BB:
-
Eup:
-
+
+
72 kD-
52 kD-
41 kD-
31 kD-
25 kD-
-
-
+
+
PDGF-BB:
-
Eup:
-
+
+
52 kD-
41 kD-
-
-
+
+
PDGF-BB:
-
Eup:
-
+
+
41 kD-
31 kD-
α-SMA
-
-
+
+
PDGF-BB:
-
Eup:
-
+
+
41 kD-
31 kD-
-
-
+
+
PDGF-BB:
-
Eup:
-
+
+
41 kD-
31 kD-
-
-
+
+
PDGF-BB:
-
Eup:
-
+
+
31 kD-
25 kD-
GAPDH
