# Supplementary figures and images for: Eupatorium lindleyanum DC Ameliorates Carbon Tetrachloride-Induced Hepatic Inflammation and Fibrotic Response in Mice
Source: Pharmaceuticals (Basel). 2025 Aug 20;18(8):1228. doi: 10.3390/ph18081228 (PMC12389491; doi:10.3390/ph18081228)

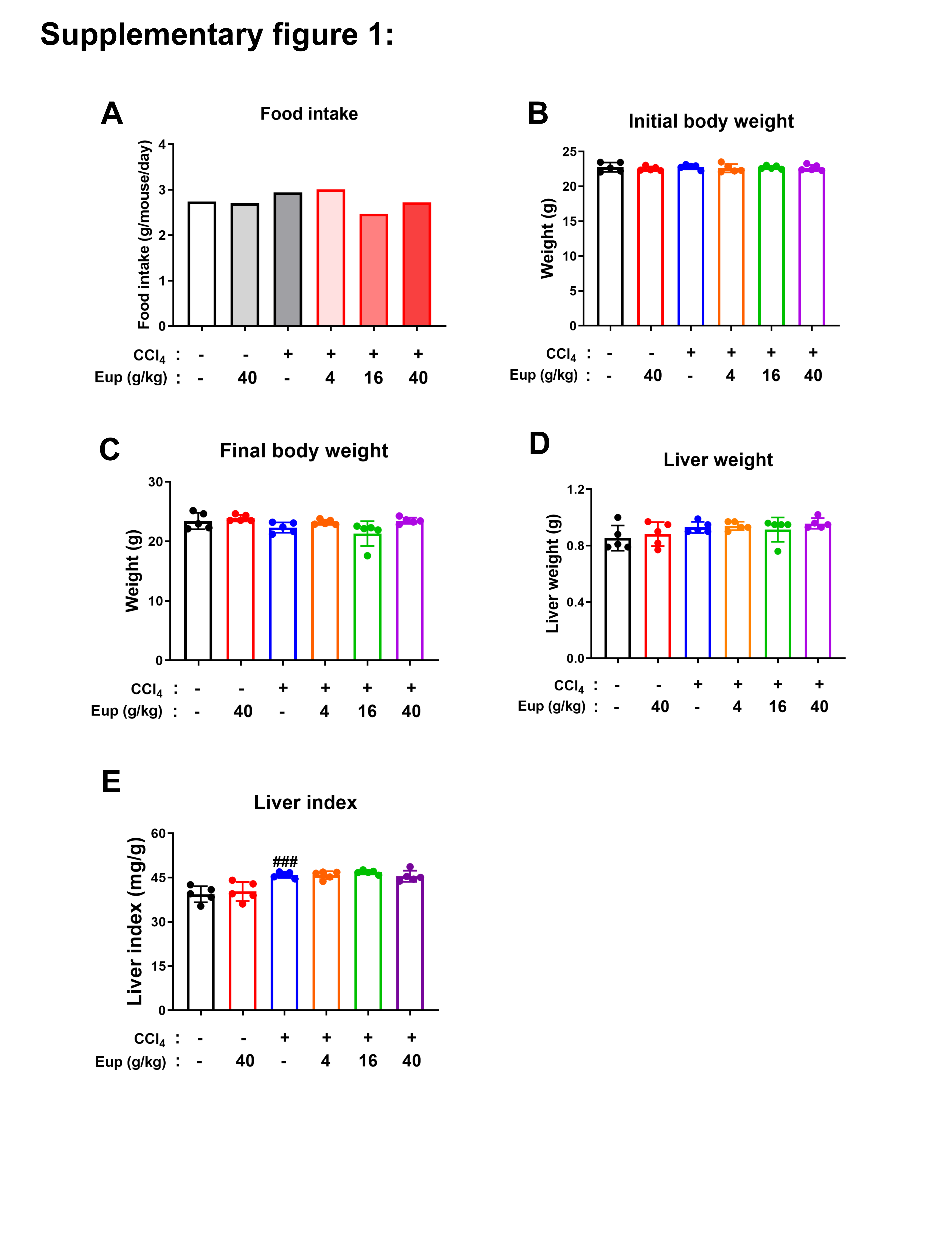

Supplement: Supplementary file 1 [file pharmaceuticals-18-01228-s001.zip › pharmaceuticals-3790117-supplementary.zip-2/supplementary figure 01.png]

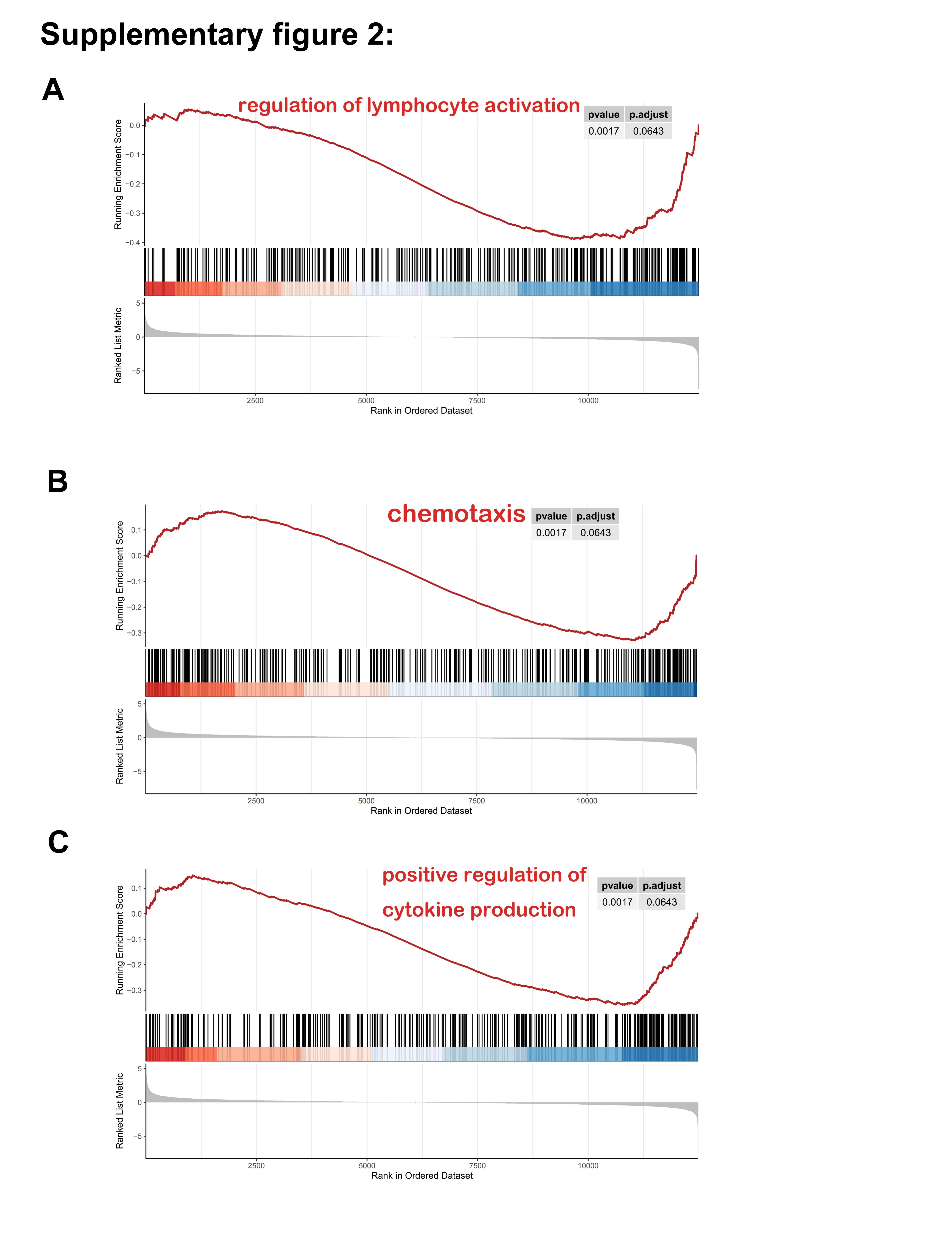

Supplement: Supplementary file 1 [file pharmaceuticals-18-01228-s001.zip › pharmaceuticals-3790117-supplementary.zip-2/supplementary figure 02.jpg]

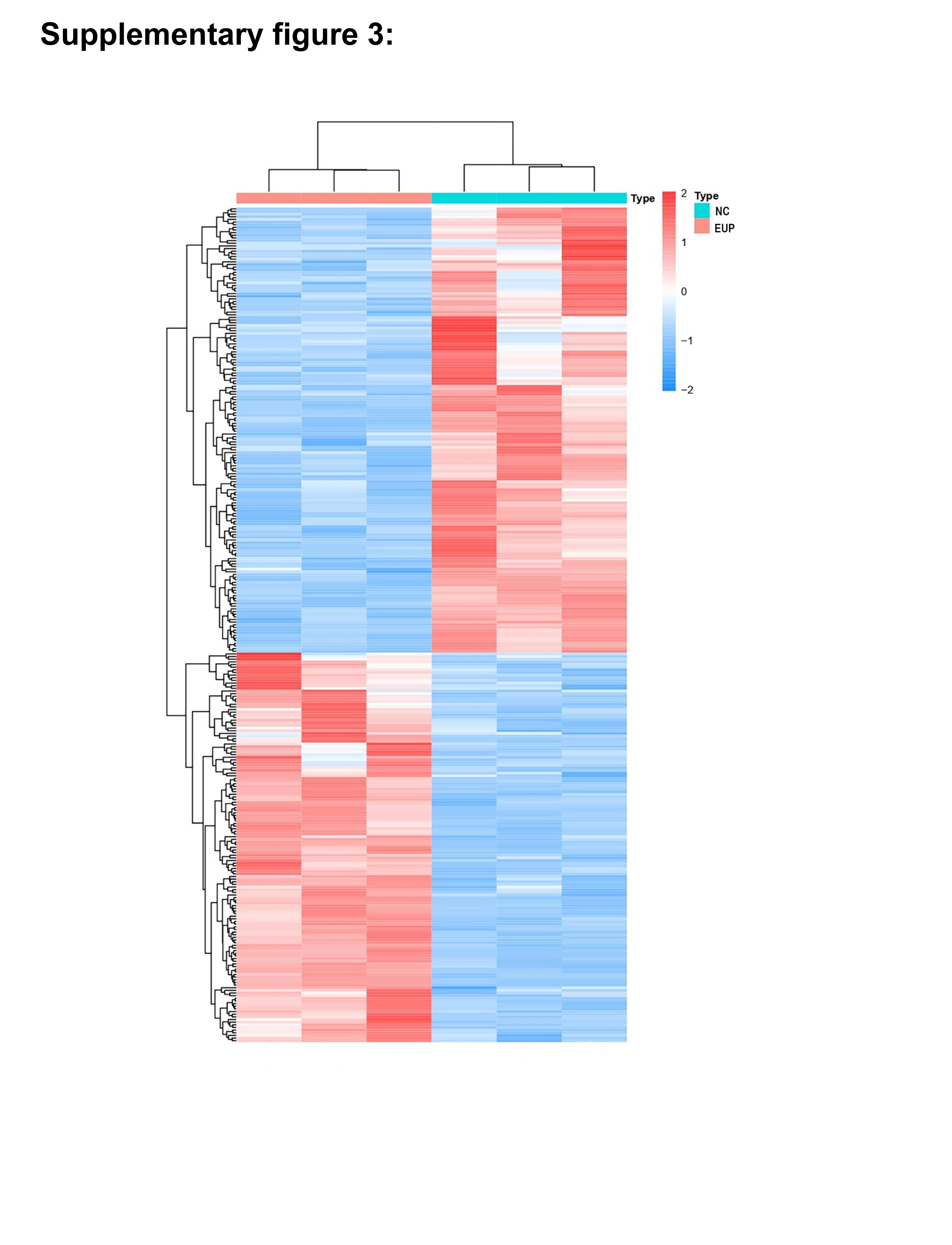

Supplement: Supplementary file 1 [file pharmaceuticals-18-01228-s001.zip › pharmaceuticals-3790117-supplementary.zip-2/supplementary figure 03.jpg]
